# Diversity in Irish and British avifauna assemblages: What can variation in diversity profiles reveal about the forces that drive assemblage composition and structure?

**DOI:** 10.1002/ece3.70143

**Published:** 2024-08-13

**Authors:** Camille Groh, Gavin M. Siriwardena, Barry J. McMahon

**Affiliations:** ^1^ UCD School of Agriculture and Food Science University College Dublin Dublin Ireland; ^2^ British Trust for Ornithology, the Nunnery Thetford Norfolk UK

**Keywords:** assemblage structure, diversity profiles, farmland birds, island biogeography

## Abstract

Ireland and Britain are two islands located at Europe's westernmost edge, both of which act as the final breeding outposts for many bird species within their European ranges. Despite their similar geographic locations and geological histories, Ireland and Britain host different breeding avifauna assemblages. Diversity profiles, which can serve as more robust alternatives to classic diversity indices, were employed in this study to explore disparities in the two islands' breeding avifauna assemblages. Variations in assemblages were explored, along with their potential drivers, through analyses at three levels: island‐scale breeding bird assemblage compositions, island‐scale diversity profiles considering 49 common breeding species, and habitat‐specific diversity profiles considering assemblages in east/central Irish farmland and East Anglian farmland. Analysis of the two islands' breeding avifauna assemblages revealed that the Irish assemblage is a complete subset of the British assemblage. Analyses of Irish and British assemblages at both an island scale and a habitat scale revealed patterns linking land use to trends within the two islands' avifauna assemblages. Irish assemblages contained greater proportions of insectivorous farmland species by abundance, while British assemblages contained greater proportions of seed‐eating farmland species; both trends appeared to be related to structural differences in agricultural land use on the two islands. The British and East Anglian assemblages exhibited higher diversity across all analyses, which appeared to be driven by the assemblages' higher relative abundances of species that were most genetically distinct. This study highlights the ability of diversity profiles to impart more information than classic diversity indices by incorporating species similarity data.

## Graphical abstract

Bird variations in assemblages were explored, along with their potential drivers, through analyses at three levels in Ireland and Britain: island‐scale breeding bird assemblage compositions, island‐scale diversity profiles considering 49 common breeding species, and habitat‐specific diversity profiles considering assemblages in east/central Irish farmland and East Anglian farmland. Analysis of the two islands' breeding avifauna assemblages revealed that the Irish assemblage is a complete subset of the British assemblage. Analyses of Irish and British assemblages at both an island scale and a habitat scale revealed patterns linking land use to trends within the two islands' avifauna assemblages.

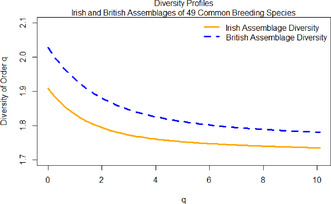

## 1 INTRODUCTION

Ireland is an 84,241 km^2^ island comprising the Republic of Ireland and Northern Ireland, which are referred to hereafter as RoI and NI, respectively. Britain is a 209,331 km^2^ island comprising England, Scotland, and Wales. The distance between the two islands ranges from 18 to 320 km (Hutchinson, 1989), and Britain is separated from mainland Europe by a distance that ranges from 34 to 580 km. Despite their similar geographic circumstances, Ireland and Britain support different breeding avifauna communities.

Previous studies indicate that Ireland's breeding avifauna assemblage contains between 137 and 157 breeding avifauna species and accounts for approximately 65%–74% of Britain's 211–213 breeding bird species (Hutchinson, 1989; Kelly et al., 2014; Smiddy, in press). Island biogeography is the most obvious driver of this variation. According to the theory of island biogeography, the number of species that an island will support is directly influenced by the island's size and its proximity to source populations. The composition of an island's fauna and flora is largely dictated by the pool of species available in its source regions (MacArthur & Wilson, 1967). Past studies indicate that both the size of British and Irish islands (including mainland Ireland and Britain) and their distance from source populations strongly influence the species richness of their breeding bird assemblages (Kelly et al., 2014). The theory of island biogeography also proposes that stepping‐stone islands facilitate biotic exchange between mainland source populations and other islands (MacArthur & Wilson, 1967). Under this assumption, Britain is a stepping‐stone between Ireland and mainland Europe. This dynamic is evidenced by the fact that at least 20 of the 23 avifauna species to successfully colonize Ireland in the last 200 years appear to have originated from British populations (Smiddy, 2014).

While island biogeography has clearly shaped British and Irish avifauna assemblages, studies suggest that variation in the two islands' assemblages exists within a Europe‐wide east–west pattern of decreasing avifauna species richness (Fuller et al., 2007; Gregory et al., 1998; Kelly et al., 2014; Tomiałojc, 2000). This further suggests that common drivers are responsible for shaping the two islands' breeding bird assemblages. This pattern has been associated with the increasing intensity and duration of human activity (Tomiałojc, 2000) and agricultural intensity (Kuemmerle et al., 2016), moving westwards across Europe.

Both Ireland and Britain were undergoing widespread deforestation by the early Bronze Age (approximately 4000 years BP); however, Britain retained a larger proportion of woodland than Ireland (Aalen et al., 1997; O'Connell & Molloy, 2001; Rackham, 2020). Human activity also resulted in both islands being dominated by agricultural habitats. Recent statistics indicate that approximately 68% of Ireland's landcover and 71% of Britain's landcover are devoted to agriculture (EPA, 2020; ONS, 2022); however, the structure of the two islands' agricultural habitats differs. Irish agriculture is dominated by grassland, with less than 8% of agricultural land being cropped (DAERA, 2021; EPA, 2020). In Britain, arable land accounts for approximately 30% of agricultural land cover, with a bias toward southern and eastern parts of the island (ONS, 2022). This marked difference in the structure of Irish and British agriculture has direct consequences for habitat availability on each island, which could explain differences in the composition and structure of Irish and British breeding bird assemblages.

While measurements of species richness can be informative, they only capture one aspect of diversity. Diversity indices integrate measures of species richness and variation in abundance, yielding more robust measurements of assemblage diversity (Magurran, 2021). Popular examples of diversity indices include the Shannon Index and the Simpson Index (Shannon, 1948; Simpson, 1949). One growing critique of such indices is their failure to consider species similarity. Diversity profiles have the potential to impart more information than classic diversity indices by incorporating species similarity data and allowing for variation in the relative influence of rare and dominant species on assemblage diversity. Similarity can refer to any biologically meaningful measure of likeness (e.g., genetic similarity, functional similarity, etc.). The profiles can be viewed as plots, which provide more context to assess assemblage diversity than the single statistics provided by classic diversity indices (Leinster & Cobbold, 2012). This method has been successfully used to study diversity in assemblages of British woodland birds where measures of similarity were based on phylogeny, habitat selection, and ecological function (Siriwardena et al., 2019). Because past comparisons of Irish and British avifauna assemblages have largely focused on species richness (Hutchinson, 1989; Kelly et al., 2014; Lack, 1969), analysing the communities through diversity profiles has the potential to illuminate finer patterns in community composition and diversity.

The core objective of this study is to compare the composition of the breeding bird assemblages in Ireland and in Britain. Assemblages are compared at both an island scale and a farmland scale using species richness data from the *Bird Atlas 2007–11: the breeding and wintering birds of Britain and Ireland* (Balmer et al., 2013), island‐scale population estimates derived from the Countryside Bird Survey (CBS) in Ireland and the Breeding Bird Survey (BBS) in Britain (Lewis et al., 2019; Musgrove et al., 2013), and data from farmland surveys performed in East Anglia and RoI (McMahon et al., 2008; Siriwardena et al., 2007). Diversity profiles were generated for assemblages at an island scale and a farmland scale, using phylogenetic data as a measure of species similarity. This study aims to answer the following questions:

1. How does breeding bird species richness differ between Ireland and Britain?
2. How do the relative abundances of farmland species included in diversity profiles compare between the two islands?
3. How do East Anglian and Irish farmland bird assemblages compare to one another, and how does this change when only species common to both assemblages are considered in analysis?
4. How do Irish and British diversity profiles, considering both island‐scale and farmland‐specific breeding bird assemblages, compare with one another?

By proposing answers to these questions, this study aims to generate hypotheses about the factors that influence fauna assemblage compositions in Ireland and Britain, using breeding birds as an example. The findings of this study can inform the context for wildlife management practices on the two islands and hence indicate whether differences in approach may be appropriate.

## 2 METHODS

### 2.1 Britain and Ireland Island‐scale breeding bird assemblage compositions and species richness values

The data used to compare breeding bird species richness in Ireland and Britain were taken from the *Bird Atlas 2007–11: the breeding and wintering birds of Britain and Ireland*, which contains distribution maps for all bird species breeding in Ireland and the UK from 2008 to 2011. Distribution maps were based on survey data from 1‐ or 2‐h timed tetrad visits (TTVs) during the four breeding seasons from 2008 to 2011. In total, 90,116 breeding season TTVs were carried out at 46,390 tetrads, each of which comprised 2 km^2^. 79,591 and 10,525 TTVs were carried out in Britain and Ireland, respectively (Balmer et al., 2013). Two species lists were compiled: one describing all Irish breeding species and one describing all British breeding species. Introduced species were excluded from the analysis. The species present on each island were then grouped by order, according to the classifications provided in the *International Ornithological Congress World Bird List* (Gill et al., 2022). The dataset and code used for this analysis can be found in appendices Table S1 and Code S2, respectively. All data analysis for this study was conducted in the program R (R Core Team, 2021).

### 2.2 Britain and Ireland Island‐scale population size data

The data on population size used to generate breeding bird diversity profiles at an island‐wide scale were taken from Musgrove et al. (2013) and Lewis et al. (2019). Breeding population estimates for the same 49 species were compiled in lists corresponding to RoI, NI, and Britain. Estimates for analogous populations in RoI and in NI were summed, yielding estimates for the island of Ireland. The Irish population estimates, along with analogous estimates for Britain, were then used to generate diversity profiles considering 49 common breeding bird species on both islands.

Breeding population estimates for RoI were collected from Lewis et al. (2019), which provided estimates for 51 of the most common breeding species in the RoI between 2011 and 2016. Population estimates were based on data from the CBS, which is an annual survey coordinated by BirdWatch Ireland and the National Parks and Wildlife Service of Ireland. Lewis et al. (2019) provided population estimates for two species that were excluded from the final analysis: swift (*Apus apus*) and pheasant (*Phasianus colchicus*). Swift was excluded due to challenges associated with attaining reliable population counts. Pheasant was excluded because it is an introduced species whose populations are typically artificially bolstered by release events.

Estimates for breeding populations of the same 49 species on the island of Britain were collected directly from Musgrove et al. (2013). Estimates for breeding populations in NI were established by subtracting Musgrove et al.'s (2013) estimates for British populations from their estimates for analogous UK populations. Population estimates provided by Musgrove et al. (2013) were based on data from the BBS. Separate British and UK population estimates were not available for five of the 49 species studied: carrion crow, stock dove (*Columba oenas*), common whitethroat (*Curruca communis*), goldfinch (*Carduelis carduelis*), and greenfinch (*Carduelis chloris*). In these cases, data from Newson et al. (2008) were used to calculate the proportion of each species that bred in NI and in Britain in 2006. These proportions were then applied to the UK population estimate given by Musgrove et al. (2013) to generate breeding population estimates for both regions.

The UK and British population estimates provided by Musgrove et al. (2013) excluded counts from the Channel Islands and most other offshore areas; however, both sets of estimates included populations located on the Isle of Man. For the 49 species whose estimates were used in this study, inclusion of the Isle of Man populations had very little impact on overall estimates (Musgrove et al., 2013). The datasets used in this analysis can be found in Table S3.

### 2.3 Ireland farmland data

**FIGURE 1:**
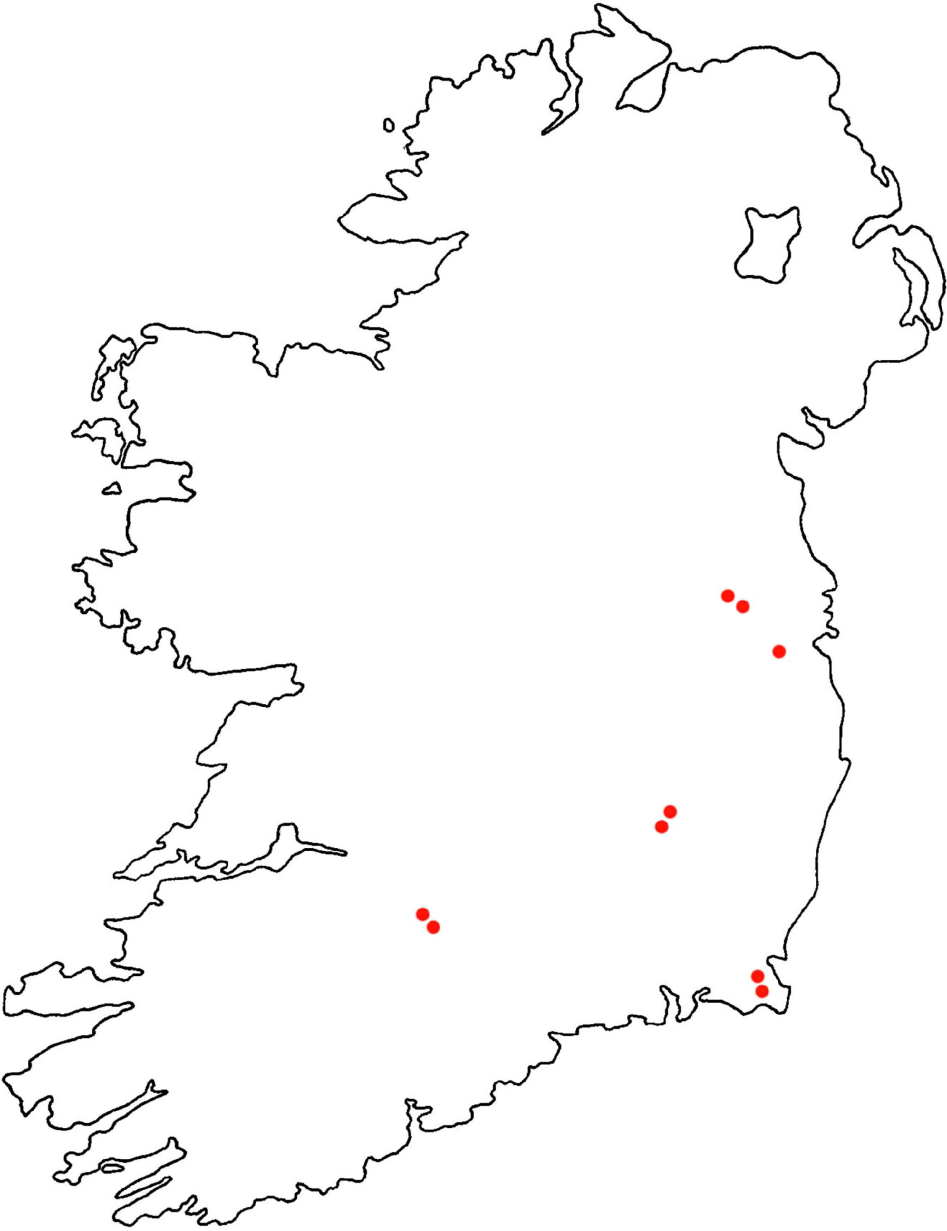
Location of farmland study sites in Ireland. Each orange point shows the general location of a site in which surveys were conducted. Sites included four dairy production sites, four mixed (arable and grassland) sites, and one beef production site.

Irish farmland survey data were derived from McMahon et al. (2008). Nine sites in eastern and central Ireland were surveyed: five research farms and four commercial farms. These nine farm sites were surveyed in the Ag‐Biota Project (Purvis et al., 2005). The farms were Lyons Estate, Teagasc Grange, Grange Commercial, Teagasc Oakpark, Oakpark Commercial, Teagasc Solohead, Solohead Commercial, Teagaasc Johnstown Castle, and Johnstown Castle Commercial. These farms form a mixture of commercial and institutional research farms (one belonging to University College Dublin and four belonging to Teagasc—the Irish agriculture and food authority and ranged in size from 18 to 87 ha, with a mean area of 54.4 ha (SD 21.8 ha)). Solohead, Johnstown, and their associated commercial sites are dairy farms, while Lyons, Oakpark, Oakpark commercial, and Grange commercial are mixed farms with arable and grassland areas. Teagasc Grange is a grassland beef production farm. Sites were surveyed four times during each of the breeding seasons (April–July) from 2003 to 2004, culminating in eight total visits to each site. During surveys, surveyors walked along field boundaries while maintaining an approximate distance of 1.5 m from field edges and speed of 2 km h^−1^ (Bibby et al., 2000). To ensure the recording of species that potentially avoid hedgerows, surveyors also walked pre‐determined transects through larger fields (Bibby et al., 2000; Chamberlain et al., 1999). All surveys commenced before 07:00 AM. When possible, farms were surveyed in their entirety. Larger farms were surveyed for approximately 3 h. Surveys were restricted to periods without persistent or heavy rain and without winds exceeding Beaufort scale 4 (WMO, 1970). Visual and aural methods were employed to detect birds. Both the presence and abundance of bird species making use of field boundaries were recorded. Raptors hunting above field boundaries were counted, but flyovers were excluded from counts (Bibby et al., 2000). Surveyors accounted for birds being flushed into other areas in order to minimize double counting (Perkins et al., 2000). The dataset used in this analysis can be found in Table S4. A map of the farmland sites surveyed is available in Figure 1.

### 2.4 British farmland data

**FIGURE 2:**
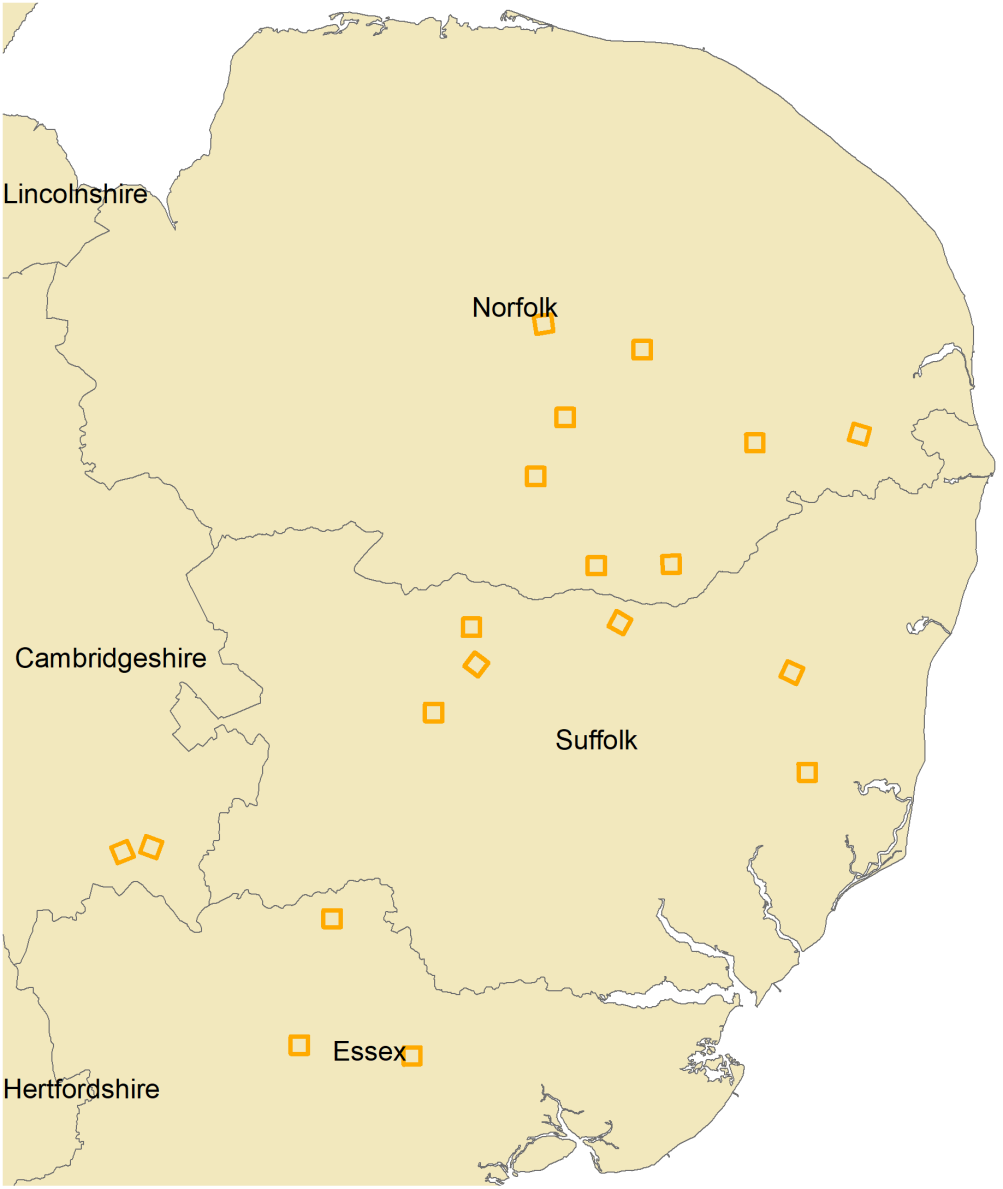
Location of East Anglian farmland study sites in the east of England. Each gold square shows a 2 × 2 km square in which surveys were conducted.

British farmland data were sourced from a follow‐up study to Siriwardena et al. (2007), using the same field methods. Twenty lowland farmland areas were surveyed in East Anglia, using the methods of the BBS. The project focused on the effects of supplementary feeding of granivorous species in farmland, but surveys were recorded across all habitats and included all species present. The farmland surveyed was dominated by tilled land with a small proportion of improved and unimproved agricultural grassland. Sites were surveyed twice during each breeding season from 2005 to 2007, culminating in six total visits to each site. Each year, one survey was completed during the early breeding season (1 April–15 May), and another survey was completed during the late breeding season (16 May–30 June). Surveys were conducted in 20 pre‐determined tetrads, comprising four 1 × 1 km^2^. Surveyors walked along two 1 km transects in each square, recording birds detected with both visual and aural methods. Birds were recorded according to habitat type (farmland, urban/built, woodland) and distance category (<25, 25–100, >100 m, and in flight). Counts commenced from 6:00 AM, and surveyors completed surveys within 4–5 h. Surveys were restricted to periods with sufficient visibility and without heavy rain or high winds. The dataset used in this analysis can be found in Table S5. A map of the farmland sites surveyed is available in Figure 2.

### 2.5 Standardization

The farmland bird survey data were standardized to allow for appropriate comparisons of Irish and East Anglian farmland bird assemblages. Because all Irish surveys were restricted to farmland habitats, any East Anglian data collected from woodland and built habitats were removed before analysis. In addition, counts of bird species dependent on waterbodies (wildfowl, gulls, terns, cormorants, kingfishers, grebes, and herons) were removed from both Irish and East Anglian survey data before analysis. These counts were removed because such species are mostly incidental to farmland bird communities, but counts of grey herons (*Ardea cinerea*) and moorhens (*Gallinula chloropus*) were retained due to evidence suggesting that both species make use of farmland ditches and ponds. Counts of pheasants and red‐legged partridges (*Alectoris rufa*) were also excluded because both species' populations are often artificially bolstered through release events.

Abundance estimates were based on the highest number of each species recorded at each site over all surveys. Each species' maximum counts were summed across all sites in Ireland and in East Anglia, respectively, generating a total count for each species encountered in each region. These totals were used as the abundance estimates in the calculation of relative abundances for diversity profiles.

### 2.6 Similarity data

Similarity matrices were constructed using phylogenetic data from a random sample of 10,000 phylogenetic trees built with the Ericson method by Jetz et al. (2012, 2014). Trees were downloaded from www.birdtree.org. Ericson trees are most likely to provide consistent results based upon simulations and comparative regression models they appear relatively robust to some degree of misspecifications in the underlying trees, either in terms of branch lengths or topology (Rubolini et al., 2015). Ericson trees were read from the Newick format and phylogenetic distances between species were calculated using the read.tree and cophenetic.phylo functions included in the ape package written for the program R (Paradis & Schliep, 2019; R Core Team, 2021). The hooded crow and the carrion crow were treated as the same species in all trees, and survey data were corrected to reflect this (Poelstra et al., 2014). The phylogenetic trees also treated the common redpoll (*Acanthis flammea*) and the lesser redpoll (*Acanthis cabaret*) as the same species, but the two species were never included in the same assemblages, so this had no impact on the analysis. A principal similarity matrix was constructed with the dimensions S × S, where S was the total number of species included in at least one of the assemblages analysed in this study. The similarity between species i and j was calculated using the equation *Z*_*ij*_ = 1 − (*d*_*ij*_/*d*_*ij*max_) where *d*_*ij*_ was the mean phylogenetic distance between species *i* and species *j* calculated for the 10,000 trees and *d*_*ij*max_ was the maximum mean distance between two species in the principal similarity matrix data. This allowed genetic distances to be standardized as a measure of similarity that ranged from 0 to 1 (Leinster & Cobbold, 2012). For each set of assemblages studied, a corresponding similarity matrix was generated by extracting the similarity data of relevant species from the principal similarity matrix.

### 2.7 Diversity profiles

Diversity profiles were constructed following the methods outlined by Leinster and Cobbold (2012). Abundance data were transformed into relative abundances where $\sum_{i=1}^{S}p_{i}=1$, where *p*_*i*_ is the relative abundance of species *i*, and where *S* is the total number of species included in the assemblage. The relative abundance of species *i* was calculated with the equation $p_{i}=a_{i}/\sum_{j=1}^{S}a_{i}$, where *a*_*i*_ describes the estimated abundance of species *i*. Similarity matrices were constructed following the methods outlined above. Following the recommendations of Leinster and Cobbold (2012), the sensitivity parameter, *q*, was tested from 0 to 10.1, using increments of 0.101. The diversity of order *q* of an assemblage was then calculated with the equation $qD^{Z}\left(p\right)={\left(\sum p_{i}{{\left(\mathrm{Zp}\right)}_{i}}^{q-1}\right)}^{1/\left(1-q\right)}$ where $\left(\mathrm{Zp}\right)i=\sum_{j=1}^{S}Z_{\mathrm{ij}}p_{j}$. Diversity profiles were plotted for each value of *q* with *q* on the *x*‐axis and the diversity of order *q* (^*q*^ *D* ^**Z**^(**p**)) on the *y*‐axis. Diversity profiles generated for corresponding assemblages were plotted alongside one another to allow for comparisons.

A single set of diversity profiles was plotted using the island scale data from Musgrove et al. (2013) and from Lewis et al. (2019). These profiles considered British and Irish assemblages of the same 49 breeding species. Two sets of diversity profiles were plotted using the farmland survey data from Ireland and East Anglia. The first set of profiles was generated with the full list of species included in the Irish and East Anglian farmland bird assemblages after standardization. An additional set of diversity profiles was plotted, using only the species recorded in both Irish and East Anglian survey data after standardization. The code used to generate diversity profiles can be found in codes S6, S7, and S8.

## 3 RESULTS

### 3.1 Breeding bird assemblage compositions

The British data comprised 209 breeding bird species, while the Irish data comprised 144 species. The Irish assemblage was a complete subset of the British assemblage and accounted for approximately 69% of Britain's breeding bird species. A breakdown of each island's breeding bird species by order is included in Table 1.

**TABLE 1:** Breeding bird species richness in Ireland and Britain grouped by order.

Order	Description	Irish species	British species	Proportional overlap
Anseriformes	Wildfowl	15	20	0.75
Galliformes	Gamebirds	3	6	0.50
Gaviiformes	Divers	1	2	0.50
Procellariformes	Albatrosses and Petrels	3	3	1.00
Pelecaniformes	Pelicans and Relatives	3	3	1.00
Ciconiiformes	Herons, Storks and Relatives	2	7	0.29
Podicipediformes	Grebes	3	4	0.75
Accippitriformes	Vultures, Hawks and Falcons	10	15	0.66
Gruiformes	Cranes, Rails, and Relatives	4	7	0.57
Charadriiformes	Shorebirds	29	39	0.74
Columbiformes	Pigeons and Doves	4	5	0.80
Cuculiformes	Cuckoos	1	1	1.00
Strigiformes	Owls	3	4	0.75
Caprimulgiformes	Nightjars and Relatives	1	1	1.00
Apodiformes	Swifts and Hummingbirds	1	1	1.00
Coraciiformes	Kingfishers and Relatives	1	1	1.00
Piciformes	Woodpeckers and Relatives	1	3	0.33
Passeriformes	Perching Birds	59	87	0.68
		144	209	0.69

*Note*: The proportional overlap column describes the proportion of species in each group included in both Islands' breeding bird assemblages. Data were gathered from the *Bird Atlas 2007–11: The breeding and wintering birds of Britain and Ireland*.

### 3.2 Diversity profiles describing 49 common breeding species at an island scale

**FIGURE 3:**
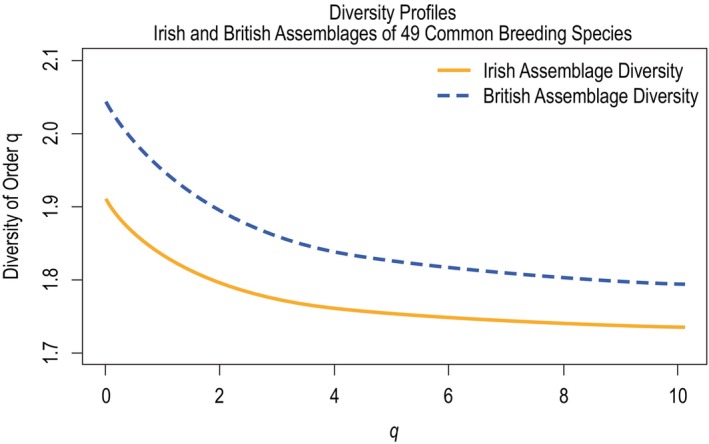
Diversity profiles considering assemblages of 48 common breeding bird species in Ireland and Britain over values of *q* from 0 to 10.1. The British assemblage exhibited higher diversity than the Irish assemblage across all values of *q*.

When diversity profiles were plotted for the two assemblages, the British assemblage exhibited higher diversity across all values of *q* (see Figure 3). This was due to the British assemblage's greater proportional abundance of species that were more genetically distinct from the bulk of the assemblage, which was dominated by passerine species. The British assemblage exhibited higher relative abundances of cuckoos, dove species, and birds of prey, all of which exhibited the highest degree of relative genetic distinctness in the assemblage's similarity matrix.

**FIGURE 4:**
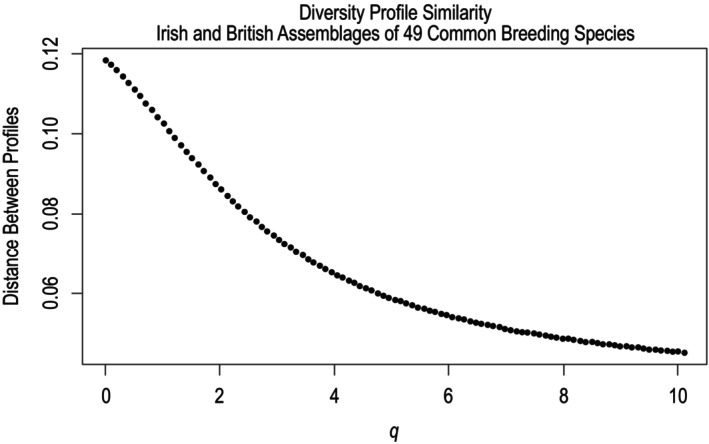
Similarity between British and Irish assemblage diversity illustrated by the vertical distance between diversity profiles over values of *q* from 0 to 10.1. The two assemblages exhibited increasingly similar diversity values as common species were given greater relative influence (as *q* increased).

Both profiles exhibited a classic diversity profile shape, displaying maximum diversity at *q* = 0 and minimum diversity at *q* = *q*_max_ (*q* = 10.1) (Leinster & Cobbold, 2012). The vertical distance between the two diversity profiles for each value of *q* is illustrated in Figure 4. This distance was decreasing across all values of *q*, suggesting diversity in the two assemblages became increasingly similar as common species were given more relative influence on assemblage diversity. The slope of the British profile was greater in magnitude than that of the Irish profile across all values of *q*. This suggests the diversity of the Irish assemblage was impacted less by changes in the influence of rare and common species on overall diversity, which is consistent with greater species evenness.

### 3.3 Diversity profiles describing farmland assemblages in Ireland and East Anglia

After standardization, a total of 80 bird species (including carrion crow and hooded crow as the same species) were recorded in Irish and East Anglian farmland survey data. A total of 50 species were recorded on Irish farmland, and 78 species were recorded on East Anglian farmland. Two species were only recorded on Irish farmland: raven (*Corvus corax*) and common redpoll (*Acanthis flammea*). Thirty species were only recorded on East Anglian farmland. Approximately 62% of the species in the East Anglian assemblage were present in the Irish assemblage, and approximately 96% of the species in the Irish assemblage were present in the East Anglian assemblage.

**FIGURE 5:**
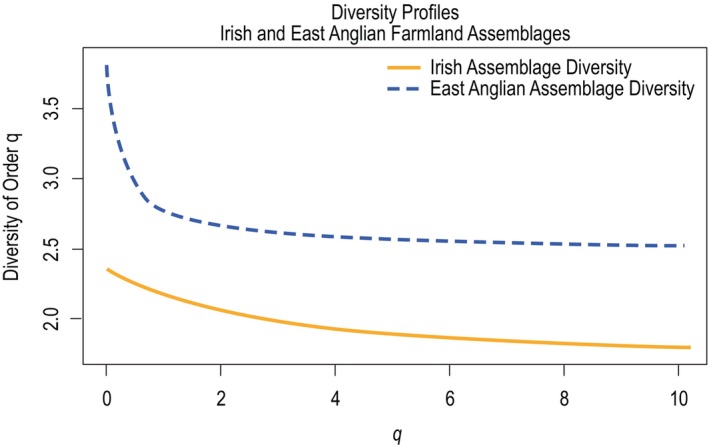
Diversity profiles considering the full standardized assemblages surveyed on Irish and East Anglian lowland farmland over values of *q* from 0 to 10.1. The East Anglian assemblage exhibited higher diversity across all values of *q*.

**FIGURE 6:**
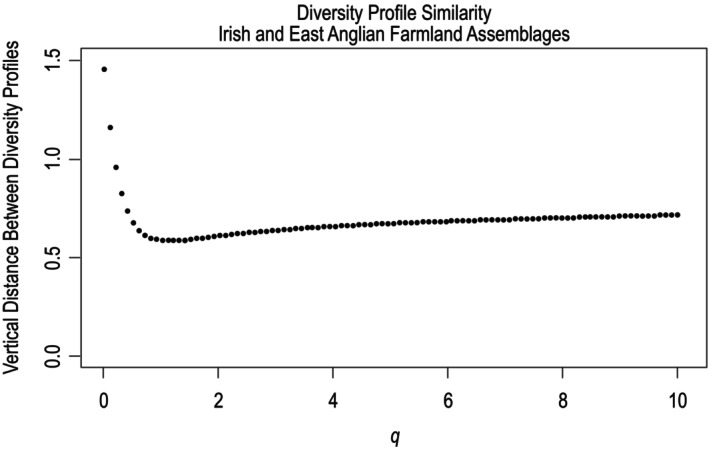
Similarity between Irish and East Anglian farmland assemblages illustrated by the vertical distance between diversity profiles over values of *q* from 0 to 10.1. The two assemblages exhibited increasing similarity in diversity from 0 < *q* < 1 and decreasing similarity from 1 < *q* < 10.1 with a maximum similarity at *q* = 1 and a minimum similarity at *q* = 0.

When diversity profiles were generated for the standardized assemblages, the East Anglian assemblage had higher diversity across all values of *q* (see Figure 5). The distance between the two profiles were decreasing for 0 < *q* ≤ 1.111 and nearly constant for 1.111 < *q* ≤ 10.1 (see Figure 6). This suggests the diversity of the two assemblages was most dissimilar when rare species and common species were considered to have the same influence on overall diversity.

**FIGURE 7:**
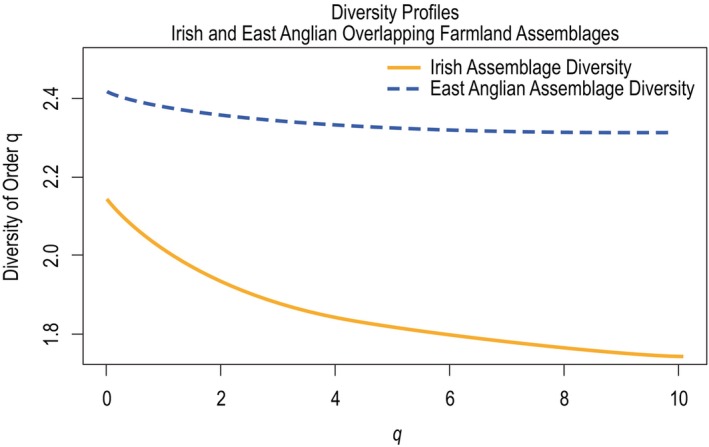
Diversity profiles considering the overlapping assemblages surveyed on Irish and East Anglian lowland farmland over values of *q* from 0 to 10.1. The East Anglian assemblage exhibited higher diversity across all values of *q*.

When assemblages of species common to the Irish and East Anglian survey data were considered, 48 species were included. When diversity profiles were generated for these assemblages, the East Anglian assemblage exhibited higher diversity across all values of *q* (see Figure 7). This was likely due to the fact that the East Anglian assemblage exhibited higher relative abundances of most of the species with the highest degrees of genetic distinctiveness (waders, raptors, and doves).

**FIGURE 8:**
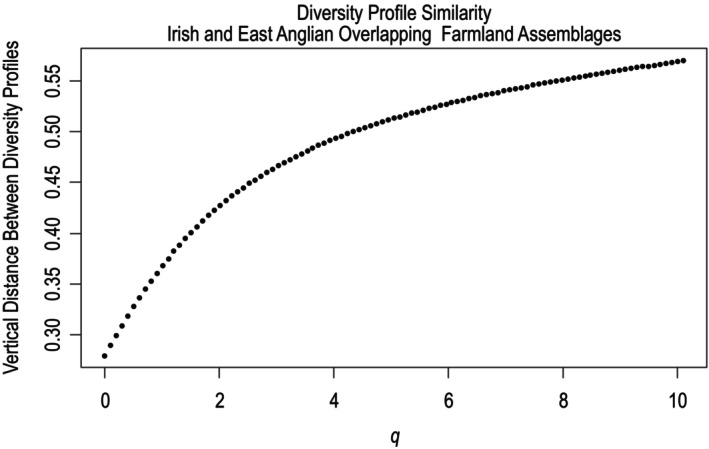
Similarity between Irish and East Anglian overlapping assemblages illustrated by the vertical distance between diversity profiles over values of *q* from 0 to 10.1. The two assemblages exhibited decreasing similarity across all values of *q*.

The two profiles were most similar when rare and common species had the same influence on diversity (at *q* = 0). The distance between the two profiles was increasing across all values of *q* (see Figure 8). The magnitude of the East Anglian profile's slope was smaller across all values of *q*. This suggests the diversity of the East Anglian assemblage was impacted less by the changes in the influence of rare and common species on overall diversity, which is consistent with greater species evenness.

## 4 DISCUSSION

### 4.1 Breeding bird assemblage compositions

When all breeding species, excluding introduced species, described by Balmer et al. (2013) were considered, the value for British breeding bird species richness was 209 and the value for Irish breeding bird species richness was 144. This value for British breeding bird species richness is slightly lower than those calculated by Hutchinson (1989) and Kelly et al. (2014), which were 211 and 213, respectively. The value for Irish breeding bird species richness is higher than the 137 species calculated by Hutchinson (1989) but lower than the 157 species calculated by Kelly et al. (2014) and the 152 species calculated by Smiddy (2014). The species richness values based on Balmer et al.'s (2013) work indicate that the Irish assemblage contains an approximate 69% of the species in the British assemblage, which falls between the 65% and 74% overlap calculated by Hutchinson (1989) and Kelly et al. (2014), respectively. The fact that all three accounts indicate that Britain supports a larger assemblage of breeding birds is consistent with MacArthur and Wilson's (1967) theory that island size positively influences species richness while distance from the mainland negatively influences species richness.

The fact that Ireland's breeding bird assemblage is a complete subset of the analogous British assemblage is consistent with island biogeography theory's concept of stepping‐stone islands, which implies Britain facilitates Ireland's colonization by acting as an intermediate area where potential source populations can become established (MacArthur & Wilson, 1967). Although the Irish assemblage is a subset of the British assemblage, there are four proposed subspecies endemic to Ireland: the Irish coal tit (*Periparus ater hibernicus*), the Irish jay (*Garrulus glandarius hibernicus*), the Irish white‐throated dipper (*Cinclus cinclus hibernicus*), and the Irish red grouse (*Lagopus lagopus hibernicus*) (Dempsey & O'Clery, 2002; Parkin & Knox, 2010). The Irish red grouse is the only proposed subspecies with basis for its treatment as a genetically distinct unit (McMahon et al., 2012; Meyer‐Lucht et al., 2016; Sangster et al., 2022). The carrion crow and hooded crow were also considered to be subspecies until recently (Poelstra et al., 2014).

### 4.2 Diversity profiles considering 49 common breeding species at an island scale

The British assemblage's diversity profile exhibited higher overall diversity due to its higher proportions of relatively genetically distinct species; however, as relatively rare species were given less influence on overall diversity, both assemblages' profiles exhibited increasingly similar diversity values. Furthermore, the Irish assemblage appeared to exhibit greater species evenness than the British assemblage. This was evidenced by the fact that the Irish assemblage's diversity profile was less sensitive to changes in the influence of rare and common species.

Twelve of the species included in the analysis are farmland birds, according to the criteria of Balmer et al. (2013), six of which exhibited higher relative abundances in the Irish assemblage and six of which exhibited higher relative abundances in the British assemblage. Variations in the relative abundances of farmland species between the Irish assemblage and the British assemblage could suggest that the different structure of agriculture on the two islands plays a role in shaping both breeding bird assemblages. The fact that five of the six farmland species that exhibited higher relative abundances in the British assemblage are granivorous is consistent with differences in the availability of cropped habitats on the two islands. Irish agriculture is dominated by grassland with less than 8% of agricultural land being cropped (DAERA, 2021; EPA, 2020) while arable land accounts for approximately 30% of British agricultural land (ONS, 2022). As a result, Ireland likely has fewer food resources to support granivorous farmland birds (Fuller et al., 1995; Gillings et al., 2005; Siriwardena et al., 1998).

Four of the six farmland species that exhibited higher relative abundances in the Irish assemblage are at least partially insectivorous during the breeding season (common whitethroat, starling, rook, and jackdaw). These results are consistent with findings that rooks, jackdaws, and starlings are positively associated with improved agricultural grasslands, likely due to higher availability of dung‐associated invertebrate food sources resulting from high stocking rates (Barnett et al., 2004; Fuller & Gough, 1999). In addition, stocking rates and fertilizer use appear to have significant positive effects on total earthworm biomass in Ireland (Muldowney et al., 2003), thereby increasing food resources for insectivorous birds.

### 4.3 Diversity profiles considering farmland assemblages in Ireland and East Anglia

The East Anglian farmland assemblage's profile exhibited higher diversity than the analogous Irish assemblage across all values of *q*. This was unsurprising given the fact that the East Anglian assemblage had a much higher species richness value (78 species) than the Irish assemblage (50 species) and a greater proportion of species that were relatively genetically distinct from the bulk of the assemblage, such as waders, raptors, owl, and doves (Leinster & Cobbold, 2012). The higher species richness observed on East Anglian farmland is consistent with MacArthur and Wilson's (1967) theory of island biogeography, which indicates that a larger island that is closer to mainland source populations (e.g., Britain) will support a greater number of species than an island that is smaller and further away from mainland source populations (e.g., Ireland). The shape of the East Anglian diversity profile, which decreases dramatically from 0 ≤ *q* ≤ 1, indicates that a relatively large proportion of the species in the assemblage were rare in the sample (Leinster & Cobbold, 2012). The approximate 62% overlap between the species encountered on East Anglian farmland and on Irish farmland was lower than the 69% overlap between species in the overall British and Irish breeding bird assemblages. This is consistent with findings that suggest the bird community in southeast Britain exhibits the least overlap with the Irish community of all the regional British bird communities (Lack, 1969).

Similar to the island‐scale analysis, when assemblages composed of species that were recorded on both East Anglian and Irish farmland were compared, the East Anglian assemblage exhibited higher diversity across all values of *q*. This was due to the higher proportions of genetically distinct species in the assemblage as well as the assemblage's higher species evenness. Unlike the island‐scale analysis, the shape of the two profiles was most similar at their left‐hand tails and least similar at their right‐hand tails. This suggests diversity values were most similar when rare species had the greatest influence (Leinster & Cobbold, 2012). The different patterns displayed at each scale suggest that the composition and structure of Irish and British breeding bird assemblages might not adhere to larger trends due to variability related to habitat and geography. Britain's arable land cover, which least resembles Ireland's agricultural grassland‐dominated landscape, is biased towards the southern and eastern parts of the island (ONS, 2022), where East Anglia is located. This probably contributes to the differences in the patterns observed in the two analyses.

Many of the trends observed in comparisons of the relative abundances of farmland species at an island scale also appeared to be present when assemblages of overlapping East Anglian and Irish farmland species were considered. According to the criteria of Balmer et al. (2013), the East Anglian assemblage contained 19 farmland specialist species while the Irish assemblage contained 14 farmland specialist species. When only overlapping species were considered, the Irish assemblage had higher relative abundances of five of the 14 farmland species included in the analysis. Four of the five farmland species with higher relative abundances in Ireland are largely insectivorous during the breeding season. Similarly, seven of the nine farmland specialists that exhibited higher relative abundances in East Anglian survey data were granivorous and/or positively associated with winter cereal stubbles (Gillings et al., 2005). These findings suggest there may be a relationship between the different structures of agriculture in East Anglia and Ireland, variations in the availability of food resources for farmland avifauna species, and the relative abundances of farmland species.

### 4.4 Limitations and considerations

The abundance data used to generate diversity profiles at an island scale were based on estimates that were subject to variation (Lewis et al., 2019; Musgrove et al., 2013). Data from Newson et al. (2008) was used to determine proportional populations of some species, which were then applied to estimates from Musgrove et al. (2013); this had the potential to conflate uncertainty in estimates. In addition, the population estimates for Britain and for Ireland are taken from two different time periods: 2009 and 2011–2016, respectively.

The methods used to survey breeding birds on Irish and East Anglian farmland differed; however, this data was standardized to allow for better comparisons (see methods section).

## 5 CONCLUSIONS

Despite sharing similar geographical locations and geological histories, Ireland and Britain support different breeding avifauna assemblages. The Irish breeding bird assemblage is a complete subset of the British assemblage, which accounts for approximately 65% of the British assemblage's total species. While island biogeography appears to be the main force driving differences between the compositions of the Irish and British breeding bird assemblages, land use dynamics and resource availability also may play a role in shaping the relative abundances of species within these assemblages as well as the overall diversity of the assemblages. Irish assemblages at an island scale and at a farmland scale generally supported higher relative abundances of insectivorous farmland avifauna species while the British and East Anglian assemblages generally supported higher relative abundances of granivorous farmland species. This trend could suggest that the different structures of agriculture on the two islands play a role in shaping their farmland assemblages. If this is the case, farmland avifauna management strategies would benefit from being developed separately for each island's assemblage of farmland species, despite the high similarity in overall species lists, thus further highlighting the challenges of the implementation of the Birds Directive 2009/147/EC. For example, while British evidence is likely to be relevant to the determination of policy measures in Ireland, the weight given to this evidence should be tempered according to the differences in ecological context. The British and East Anglian assemblages exhibited higher diversity across all analyses. This appeared to be driven by the assemblages' higher relative abundances of species that were most genetically distinct. Notably, this would not be captured by classic diversity indices, which do not incorporate genetic similarity into diversity value calculations. This study highlights the ability of diversity profiles to impart more information than classic diversity indices by incorporating species similarity data.

## AUTHOR CONTRIBUTIONS

**Camille Groh:** Formal analysis (lead); methodology (lead); writing – original draft (lead). **Gavin M. Siriwardena:** Conceptualization (equal); data curation (equal); formal analysis (supporting); methodology (supporting); writing – review and editing (equal). **Barry J. McMahon:** Conceptualization (equal); data curation (equal); formal analysis (supporting); methodology (supporting); project administration (lead); supervision (lead); writing – review and editing (equal).

## CONFLICT OF INTEREST STATEMENT

The authors declare they have no conflict of interest.

## Supporting information


Data S1.

## Data Availability

All data from this paper are made available via the supporting material.
