# Supplementary material for: Diversity in Irish and British avifauna assemblages: What can variation in diversity profiles reveal about the forces that drive assemblage composition and structure?
Source: Ecol Evol. 2024 Aug 13;14(8):e70143. doi: 10.1002/ece3.70143 (PMC11319734; doi:10.1002/ece3.70143)
Supplement: Supplementary file 1 — Data S1. [file ECE3-14-e70143-s001.docx]

**Supplementary Material**

Table S1: Breeding bird species in Ireland and in Britain

Code S2: Analysis of Irish and British breeding bird species richness

Table S3: Breeding population estimates for 49 species in RoI, NI and Britain

Table S4: Irish farmland count data

Table S5: East Anglian farmland count data

Code S6: Diversity profiles considering breeding bird assemblages in Ireland and Britain

Code S7: Diversity profiles considering breeding bird assemblages on Irish and East Anglian farmland

Code S8: Diversity profiles considering breeding bird assemblages of overlapping species on Irish and East Anglian farmland

Table S1: Breeding bird species in Ireland and in Britain. Species that appear in further analyses are marked in bold.

| Order | Common | Latin | Ireland | Britain |
| --- | --- | --- | --- | --- |
| Wildfowl | Mute swan | *Cygnus olor* | Present | Present |
| Wildfowl | Whooper swan | *Cygnus cygnus* | Present | Present |
| Wildfowl | Pink-footed goose | *Anser brachyrhynchus* | Absent | Present |
| Wildfowl | Greylag goose | *Anser anser* | Present | Present |
| Wildfowl | Shelduck | *Tadorna tadorna* | Present | Present |
| Wildfowl | Wigeon | *Anas penelope* | Absent | Present |
| Wildfowl | Gadwall | *Mareca strepera* | Present | Present |
| Wildfowl | Teal | *Anas crecca* | Present | Present |
| Wildfowl | Mallard | *Anas platyrhynchos* | Present | Present |
| Wildfowl | Pintail | *Anas acuta* | Absent | Present |
| Wildfowl | Garganey | *Anas querquedula* | Present | Present |
| Wildfowl | Shoveler | *Spatula clypeata* | Present | Present |
| Wildfowl | Red-crested pochard | *Netta rufina* | Absent | Present |
| Wildfowl | Pochard | *Aythya ferina* | Present | Present |
| Wildfowl | Tufted duck | *Aythya fuligula* | Present | Present |
| Wildfowl | Eider | *Somateria mollissima* | Present | Present |
| Wildfowl | Common scoter | *Melanitta nigra* | Present | Present |
| Wildfowl | Goldeneye | *Bucephala clangula* | Absent | Present |
| Wildfowl | Red-breasted merganser | *Mergus serrator* | Present | Present |
| Wildfowl | Goosander | *Mergus merganser* | Present | Present |
| Gamebirds | Quail | *Coturnix coturnix* | Present | Present |
| Gamebirds | Red grouse | *Lagopus lagopus* | Present | Present |
| Gamebirds | Ptarmigan | *Lagopus muta* | Absent | Present |
| Gamebirds | Black grouse | *Lyrurus tetrix* | Absent | Present |
| Gamebirds | Capercaillie | *Tetrao urogallus* | Absent | Present |
| **Gamebirds** | **Grey partridge** | ***Perdix perdix*** | **Present** | **Present** |
| Divers | Red-throated diver | *Gavia stellata* | Present | Present |
| Divers | Black-throated diver | *Gavia arctica* | Absent | Present |
| Albatrosses and Petrels | Fulmar | *Fulmaris glacialis* | Present | Present |
| Albatrosses and Petrels | Manx shearwater | *Puffinus puffinus* | Present | Present |
| Albatrosses and Petrels | Storm petrel | *Hydrobates pelagicus* | Present | Present |
| Pelicans and Relatives | Gannet | *Morus bassanus* | Present | Present |
| Pelicans and Relatives | Cormorant | *Phalacrocorax carbo* | Present | Present |
| Pelicans and Relatives | Shag | *Gulosus aristotelis* | Present | Present |
| Herons, storks, and relatives | Bittern | *Botaurus stellaris* | Absent | Present |
| Herons, storks, and relatives | Little bittern | *Ixobrychus minutus* | Absent | Present |
| Herons, storks, and relatives | Cattle egret | *Bubulcus ibis* | Absent | Present |
| Herons, storks, and relatives | Little egret | *Egretta garzetta* | Present | Present |
| **Herons, storks, and relatives** | **Grey heron** | ***Ardea cinerea*** | **Present** | **Present** |
| Herons, storks, and relatives | Purple heron | *Ardea purpurea* | Absent | Present |
| Herons, storks, and relatives | Spoonbill | *Platalea leucorodia* | Absent | Present |
| Grebes | Little grebe | *Tachybaptus ruficollis* | Present | Present |
| Grebes | Great crested grebe | *Podiceps cristatus* | Present | Present |
| Grebes | Slavonian grebe | *Podiceps auritus* | Absent | Present |
| Grebes | Black-necked grebe | *Podiceps nigricollis* | Present | Present |
| Vultures, hawks, falcons | Red kite | *Milvus milvus* | Present | Present |
| Vultures, hawks, falcons | White-tailed eagle | *Haliaeetus albicilla* | Absent | Present |
| **Vultures, hawks, falcons** | **Marsh harrier** | ***Circus aeruginosis*** | **Present** | **Present** |
| Vultures, hawks, falcons | Hen harrier | *Circus cyaneus* | Present | Present |
| Vultures, hawks, falcons | Montagu's harrier | *Circus pygargus* | Absent | Present |
| Vultures, hawks, falcons | Honey buzzard | *Pernis apivorus* | Absent | Present |
| Vultures, hawks, falcons | Goshawk | *Accipiter gentilis* | Present | Present |
| **Vultures, hawks, falcons** | **Sparrowhawk** | ***Accipiter nisus*** | **Present** | **Present** |
| **Vultures, hawks, falcons** | **Buzzard** | ***Buteo buteo*** | **Present** | **Present** |
| Vultures, hawks, falcons | Golden eagle | *Aquila chrysaetos* | Present | Present |
| Vultures, hawks, falcons | Osprey | *Pandion haliaetus* | Absent | Present |
| **Vultures, hawks, falcons** | **Kestrel** | ***Falco tinnunculus*** | **Present** | **Present** |
| Vultures, hawks, falcons | Merlin | *Falco columbarius* | Present | Present |
| **Vultures, hawks, falcons** | **Hobby** | ***Falco subbuteo*** | **Absent** | **Present** |
| Vultures, hawks, falcons | Peregrine falcon | *Falco peregrinus* | Present | Present |
| Cranes, rails, and relatives | Water rail | *Rallus aquaticus* | Present | Present |
| Cranes, rails, and relatives | Spotted crake | *Porzana porzana* | Absent | Present |
| Cranes, rails, and relatives | Corncrake | *Crex crex* | Present | Present |
| **Cranes, rails, and relatives** | **Moorhen** | ***Gallinula chloropus*** | **Present** | **Present** |
| Cranes, rails, and relatives | Coot | *Fulica atra* | Present | Present |
| Cranes, rails, and relatives | Crane | *Grus grus* | Absent | Present |
| Cranes, rails, and relatives | Great bustard | *Otis tarda* | Absent | Present |
| Shorebirds | Black-winged stilt | *Himantopus himantopus* | Absent | Present |
| **Shorebirds** | **Oystercatcher** | ***Haematopus ostralegus*** | **Present** | **Present** |
| Shorebirds | Avocet | *Recurvirostra avosetta* | Absent | Present |
| Shorebirds | Stone curlew | *Burhinus oedicnemus* | Absent | Present |
| Shorebirds | Little ringed plover | *Charadrius dubius* | Present | Present |
| Shorebirds | Ringed plover | *Charadrius hiaticula* | Present | Present |
| Shorebirds | Dotterel | *Charadrius morinellus* | Absent | Present |
| **Shorebirds** | **Golden plover** | ***Pluvialis apricaria*** | **Present** | **Present** |
| **Shorebirds** | **Lapwing** | ***Vanellus vanellus*** | **Present** | **Present** |
| Shorebirds | Purple sandpiper | *Calidris maritima* | Absent | Present |
| Shorebirds | Dunlin | *Calidris alpina* | Present | Present |
| **Shorebirds** | **Snipe** | ***Gallinago gallinago*** | **Present** | **Present** |
| Shorebirds | Woodcock | *Scolopax rusticola* | Present | Present |
| Shorebirds | Black-tailed godwit | *Limosa limosa* | Present | Present |
| **Shorebirds** | **Curlew** | ***Numenius arquata*** | **Present** | **Present** |
| Shorebirds | Common sandpiper | *Actitis hypoleucos* | Present | Present |
| Shorebirds | Green sandpiper | *Tringa ochropus* | Absent | Present |
| Shorebirds | Wood sandpiper | *Tringa glareola* | Absent | Present |
| Shorebirds | Greenshank | *Tringa nebularia* | Absent | Present |
| Shorebirds | Redshank | *Tringa totanus* | Present | Present |
| Shorebirds | Arctic skua | *Stercorarius parasiticus* | Absent | Present |
| Shorebirds | Great skua | *Stercorarius skua* | Present | Present |
| Shorebirds | Kittiwake | *Rissa tridactyla* | Present | Present |
| Shorebirds | Black-headed gull | *Chroicocephalus ridibundus* | Present | Present |
| Shorebirds | Mediterranean gull | *Larus melanocephalus* | Present | Present |
| Shorebirds | Common gull | *Larus canus* | Present | Present |
| Shorebirds | Lesser black-backed gull | *Larus fuscus* | Present | Present |
| Shorebirds | Herring gull | *Larus argentatus* | Present | Present |
| Shorebirds | Yellow-legged gull | *Larus michahellis* | Absent | Present |
| Shorebirds | Great black-backed gull | *Larus marinus* | Present | Present |
| Shorebirds | Little tern | *Sternula albifrons* | Present | Present |
| Shorebirds | Sandwich tern | *Sternula sandvicensis* | Present | Present |
| Shorebirds | Common tern | *Sterna hirundo* | Present | Present |
| Shorebirds | Arctic tern | *Sterna paradisaea* | Present | Present |
| Shorebirds | Roseate tern | *Sterna dougallii* | Present | Present |
| Shorebirds | Guillemot | *Uria aalge* | Present | Present |
| Shorebirds | Razorbill | *Alca torda* | Present | Present |
| Shorebirds | Black guillemot | *Cepphus grylle* | Present | Present |
| Shorebirds | Puffin | *Fratercula arctica* | Present | Present |
| **Pigeons and doves** | **Rock dove/feral pigeon** | ***Columba livia*** | **Present** | **Present** |
| **Pigeons and doves** | **Stock dove** | ***Columba oenas*** | **Present** | **Present** |
| **Pigeons and doves** | **Wood pigeon** | ***Columba palumbus*** | **Present** | **Present** |
| **Pigeons and doves** | **Collared dove** | ***Streptopelia decaocto*** | **Present** | **Present** |
| **Pigeons and doves** | **Turtle dove** | ***Streptopelia turtur*** | **Absent** | **Present** |
| **Cuckoos** | **Cuckoo** | ***Cuculus canorus*** | **Present** | **Present** |
| **Owls** | **Barn owl** | ***Tyto alba*** | **Present** | **Present** |
| **Owls** | **Tawny owl** | ***Strix aluco*** | **Absent** | **Present** |
| **Owls** | **Long-eared owl** | ***Asio otus*** | **Present** | **Present** |
| Owls | Short-eared owl | *Asio flammeus* | Present | Present |
| Nightjars and relatives | Nightjar | *Caprimulgus europaeus* | Present | Present |
| **Swifts and hummingbirds** | **Swift** | ***Apus apus*** | **Present** | **Present** |
| Kingfishers and allies | Kingfisher | *Alcedo athis* | Present | Present |
| **Woodpeckers and allies** | **Green woodpecker** | ***Picus viridis*** | **Absent** | **Present** |
| **Woodpeckers and allies** | **Greatspotted woodpecker** | ***Dendrocopos major*** | **Present** | **Present** |
| Woodpeckers and allies | Lesser-spotted woodpecker | *Dendrocopos minor* | Absent | Present |
| Perching birds | Golden oriole | *Oriolus oriolus* | Absent | Present |
| Perching birds | Red-backed shrike | *Lanius collurio* | Absent | Present |
| Perching birds | Chough | *Pyrrhocorax pyrrhocorax* | Present | Present |
| **Perching birds** | **Magpie** | ***Pica pica*** | **Present** | **Present** |
| **Perching birds** | **Jay** | ***Garrulus glandarius*** | **Present** | **Present** |
| **Perching birds** | **Jackdaw** | ***Corvus monedula*** | **Present** | **Present** |
| **Perching birds** | **Rook** | ***Corvus frugilegus*** | **Present** | **Present** |
| **Perching birds** | **Carrion crow** | ***Corvus corone*** | **Present** | **Present** |
| **Perching birds** | **Hooded crow** | ***Corvus cornix*** | **Present** | **Present** |
| **Perching birds** | **Raven** | ***Corvus corax*** | **Present** | **Present** |
| **Perching birds** | **Goldcrest** | ***Regulus regulus*** | **Present** | **Present** |
| **Perching birds** | **Firecrest** | ***Regulus ignicapilla*** | **Absent** | **Present** |
| **Perching birds** | **Blue tit** | ***Cyanistes caeruleus*** | **Present** | **Present** |
| **Perching birds** | **Great tit** | ***Parus major*** | **Present** | **Present** |
| Perching birds | Crested tit | *Lophophanes cristatus* | Absent | Present |
| **Perching birds** | **Coal tit** | ***Periparus ater*** | **Present** | **Present** |
| Perching birds | Willow tit | *Poecile montana* | Absent | Present |
| **Perching birds** | **Marsh tit** | ***Poecile palustris*** | **Absent** | **Present** |
| Perching birds | Bearded tit | *Panurus biarmicus* | Present | Present |
| Perching birds | Woodlark | *Lullula arborea* | Absent | Present |
| **Perching birds** | **Skylark** | ***Alauda arvensis*** | **Present** | **Present** |
| **Perching birds** | **Sand martin** | ***Riparia riparia*** | **Present** | **Present** |
| **Perching birds** | **Swallow** | ***Hirundo rustica*** | **Present** | **Present** |
| **Perching birds** | **House martin** | ***Delichon urbicum*** | **Present** | **Present** |
| Perching birds | Cetti's warbler | *Cetti cetti* | Absent | Present |
| **Perching birds** | **Long-tailed tit** | ***Aegithalos caudatus*** | **Present** | **Present** |
| Perching birds | Wood warbler | *Phylloscopus sibilatrix* | Present | Present |
| **Perching birds** | **Chiffchaff** | ***Phylloscopus collybita*** | **Present** | **Present** |
| **Perching birds** | **Willow warbler** | ***Phylloscopus trochilus*** | **Present** | **Present** |
| **Perching birds** | **Blackcap** | ***Sylvia atricapilla*** | **Present** | **Present** |
| **Perching birds** | **Garden warbler** | ***Sylvia borin*** | **Present** | **Present** |
| **Perching birds** | **Lesser whitethroat** | ***Sylvia curruca*** | **Absent** | **Present** |
| **Perching birds** | **Whitethroat** | ***Sylvia communis*** | **Present** | **Present** |
| Perching birds | Dartford warbler | *Sylvia undata* | Absent | Present |
| **Perching birds** | **Grasshopper warbler** | ***Locustella naevia*** | **Present** | **Present** |
| **Perching birds** | **Sedge warbler** | ***Acrocephalus schoenobaenus*** | **Present** | **Present** |
| **Perching birds** | **Reed warbler** | ***Acrocephalus scirpaceus*** | **Present** | **Present** |
| Perching birds | Marsh warbler | *Acrocephalus palustris* | Absent | Present |
| Perching birds | Savi's warbler | *Locustella luscinioides* | Absent | Present |
| Perching birds | Icterine warbler | *Hippolais icterina* | Absent | Present |
| **Perching birds** | **Nuthatch** | ***Sitta europaea*** | **Absent** | **Present** |
| **Perching birds** | **Treecreeper** | ***Certhia familiaris*** | **Present** | **Present** |
| **Perching birds** | **Wren** | ***Troglodytes troglodytes*** | **Present** | **Present** |
| **Perching birds** | **Starling** | ***Sterling vulgaris*** | **Present** | **Present** |
| Perching birds | Dipper | *Cinclus cinclus* | Present | Present |
| Perching birds | Ring ouzel | *Turdus torquatus* | Present | Present |
| **Perching birds** | **Blackbird** | ***Turdus merula*** | **Present** | **Present** |
| **Perching birds** | **Fieldfare** | ***Turdus pilaris*** | **Absent** | **Present** |
| **Perching birds** | **Song thrush** | ***Turdus philomelos*** | **Present** | **Present** |
| Perching birds | Redwing | *Turdus iliacus* | Absent | Present |
| **Perching birds** | **Mistle thrush** | ***Turdus Viscivorus*** | **Present** | **Present** |
| **Perching birds** | **Spotted flycatcher** | ***Muscicapa striata*** | **Present** | **Present** |
| **Perching birds** | **Robin** | ***Erithacus rubecula*** | **Present** | **Present** |
| **Perching birds** | **Nightingale** | ***Luscinia megarhynchos*** | **Absent** | **Present** |
| Perching birds | Pied flycatcher | *Ficedula hypoleuca* | Absent | Present |
| Perching birds | Black redstart | *Phoenicurus ochrucos* | Absent | Present |
| Perching birds | Redstart | *Phoenicurus phoenicurus* | Present | Present |
| Perching birds | Whinchat | *Saxicola rubetra* | Present | Present |
| **Perching birds** | **Stonechat** | ***Saxicola rubicola*** | **Present** | **Present** |
| **Perching birds** | **Wheatear** | ***Oenanthe oenanthe*** | **Present** | **Present** |
| **Perching birds** | **Dunnock** | ***Prunella modularis*** | **Present** | **Present** |
| **Perching birds** | **House sparrow** | ***Passer domesticus*** | **Present** | **Present** |
| **Perching birds** | **Tree sparrow** | ***Passer montanus*** | **Present** | **Present** |
| **Perching birds** | **Yellow wagtail** | ***Motacilla flava*** | **Absent** | **Present** |
| **Perching birds** | **Grey wagtail** | ***Motacilla cinerea*** | **Present** | **Present** |
| **Perching birds** | **Pied wagtail** | ***Motacilla alba*** | **Present** | **Present** |
| Perching birds | Tree pipit | *Anthus trivialis* | Absent | Present |
| **Perching birds** | **Meadow pipit** | ***Anthus pratensis*** | **Present** | **Present** |
| Perching birds | Rock pipit | *Anthus petrosus* | Present | Present |
| **Perching birds** | **Chaffinch** | ***Fringilla coelebs*** | **Present** | **Present** |
| **Perching birds** | **Greenfinch** | ***Chloris chloris*** | **Present** | **Present** |
| **Perching birds** | **Goldfinch** | ***Carduelis carduelis*** | **Present** | **Present** |
| Perching birds | Siskin | *Carduelis spinus* | Present | Present |
| **Perching birds** | **Linnet** | ***Carduelis cannabina*** | **Present** | **Present** |
| Perching birds | Twite | *Carduelis flavirostros* | Present | Present |
| **Perching birds** | **Lesser redpoll** | ***Carduelis cabaret*** | **Present** | **Present** |
| **Perching birds** | **Common redpoll** | ***Carduelis flammea*** | **Absent** | **Present** |
| Perching birds | Common crossbill | *Loxia curvirostra* | Present | Present |
| Perching birds | Scottish crossbill | *Loxia scotica* | Absent | Present |
| Perching birds | Parrot crossbill | *Loxia pytyopsittacus* | Absent | Present |
| **Perching birds** | **Bullfinch** | ***Pyrrhula pyrrhula*** | **Present** | **Present** |
| Perching birds | Hawfinch | *Coccothraustes coccothraustes* | Absent | Present |
| Perching birds | Snow bunting | *Plectrophenax nivalis* | Absent | Present |
| **Perching birds** | **Yellowhammer** | ***Emberiza citrinella*** | **Present** | **Present** |
| Perching birds | Cirl bunting | *Emberiza cirlus* | Absent | Present |
| **Perching birds** | **Reed bunting** | ***Emberiza schoeniclus*** | **Present** | **Present** |
| **Perching birds** | **Corn bunting** | ***Emberiza calandra*** | **Absent** | **Present** |

Code S2: Analysis of Irish and British breeding bird species richness

---

title: "Species Richness"

author: "Camille Groh"

output: html_document

---

```{r}

sr <- read.csv("sr.csv", header = TRUE, sep = ",")

##make presence/absence binary

for (i in 1:length(sr$Ireland)) {

if(sr$Ireland[i] == "Present") {

sr$Ireland[i] <- 1

} else

sr$Ireland[i] <- 0

}

for (i in 1:length(sr$Britain)) {

if(sr$Britain[i] == "Present") {

sr$Britain[i] <- 1

} else

sr$Britain[i] <- 0

}

##transform to numeric data

sr$Ireland <- as.numeric(sr$Ireland)

sr$Britain <- as.numeric(sr$Britain)

```

#analysis

```{r}

##total sr

irelandsr <- sum(sr$Ireland)

gbsr <- sum(sr$Britain)

##subset by order in a list

orders <- unique(sr$Order)

orderlist <- list()

for (i in 1:length(orders)) {

orderlist[[i]] <- subset(sr, sr$Order == orders[i])

}

##calculate sr by order

ieordertotals <- c()

gbordertotals <- c()

for (i in 1:length(orders)) {

ieordertotals <- c(ieordertotals, sum(orderlist[[i]]$Ireland))

gbordertotals <- c(gbordertotals, sum(orderlist[[i]]$Britain))

}

names(ieordertotals) <- orders

names(gbordertotals) <- orders

ieordertotals/gbordertotals

```

Table S3: Breeding population estimates for 49 species in RoI, NI and Britain

| Species Common Name | RoI | NI | Britain |
| --- | --- | --- | --- |
| Collared Dove | 260939 | 10000 | 980000 |
| Stock Dove | 27486 | 0 | 260000 |
| Wood Pigeon | 2573909 | 100000 | 5300000 |
| Sparrowhawk | 11859 | 2000 | 33000 |
| Kestrel | 13500 | 1000 | 45000 |
| Magpie | 563536 | 50000 | 550000 |
| Jackdaw | 2564415 | 100000 | 1300000 |
| Raven | 55878 | 400 | 7000 |
| Rook | 3476836 | 110000 | 990000 |
| Carrion Crow | 523293 | 100000 | 1160000 |
| Coal Tit | 836424 | 80000 | 680000 |
| Great Tit | 1288058 | 100000 | 2500000 |
| Blue tit | 1942762 | 200000 | 3400000 |
| Skylark | 301800 | 100000 | 1400000 |
| Long-tailed Tit | 101834 | 10000 | 330000 |
| Willow Warbler | 1721483 | 200000 | 2200000 |
| Chiffchaff | 345748 | 100000 | 1100000 |
| Swallow | 4936488 | 100000 | 760000 |
| House Martin | 606043 | 5000 | 510000 |
| Sand Martin | 460223 | 10000 | 104000 |
| Whitethroat | 97099 | 4000 | 1096000 |
| Black Cap | 586216 | 100000 | 1100000 |
| Grasshopper  Warbler | 22382 | 3000 | 13000 |
| Sedge Warbler | 115726 | 30000 | 260000 |
| Starling | 2066904 | 100000 | 1800000 |
| Mistle Thrush | 185200 | 10000 | 160000 |
| Song Thrush | 853569 | 100000 | 1100000 |
| Blackbird | 4613945 | 200000 | 4900000 |
| Robin | 4517711 | 700000 | 6000000 |
| Wheatear | 43635 | 10000 | 230000 |
| Stonechat | 93088 | 3000 | 56000 |
| Spotted Flycatcher | 34197 | 3000 | 33000 |
| Treecreeper | 77344 | 20000 | 180000 |
| Wren | 5552467 | 900000 | 7700000 |
| Goldcrest | 601806 | 90000 | 520000 |
| Linnet | 459892 | 20000 | 410000 |
| Goldfinch | 1107425 | 20000 | 1180000 |
| Lesser Redpoll | 358973 | 30000 | 190000 |
| Greenfinch | 536730 | 30000 | 1670000 |
| Bullfinch | 648630 | 30000 | 190000 |
| Chaffinch | 3690474 | 400000 | 5800000 |
| Reed Bunting | 191922 | 20000 | 230000 |
| Yellowhammer | 217252 | 10000 | 700000 |
| Grey Wagtail | 50768 | 3000 | 35000 |
| Pied Wagtail | 501741 | 10000 | 460000 |
| Meadow Pipit | 1351995 | 200000 | 1900000 |
| House Sparrow | 2266646 | 200000 | 5100000 |
| Dunnock | 1630331 | 200000 | 2300000 |
| Cuckoo | 11808 | 1000 | 15000 |

|  |  |
| --- | --- |

Table S4: Irish farmland count data. Sites one is a beef production; sites two, five, six and seven are mixed farmlands with arable and grassland areas; sites three, four, eight and nine are dairy productions.

|  | Site1 | Site2 | Site3 | Site4 | Site5 | Site6 | Site7 | Site8 | Site9 |
| --- | --- | --- | --- | --- | --- | --- | --- | --- | --- |
| Sparrowhawk | 0 | 0 | 0 | 1 | 0 | 0 | 0 | 0 | 0 |
| Moorhen | 0 | 1 | 1 | 1 | 0 | 0 | 0 | 0 | 0 |
| Snipe | 1 | 0 | 0 | 0 | 0 | 0 | 0 | 0 | 0 |
| Stock Dove | 0 | 0 | 0 | 0 | 2 | 3 | 0 | 0 | 0 |
| Wood Pigeon | 24 | 24 | 10 | 8 | 20 | 105 | 15 | 10 | 10 |
| Collared Dove | 0 | 0 | 0 | 6 | 0 | 0 | 0 | 0 | 0 |
| Skylark | 0 | 0 | 0 | 0 | 2 | 13 | 7 | 1 | 0 |
| Meadow Pipit | 1 | 0 | 6 | 5 | 2 | 3 | 0 | 6 | 0 |
| Pied Wagtail | 0 | 2 | 3 | 2 | 5 | 1 | 0 | 2 | 1 |
| Wren | 22 | 24 | 17 | 13 | 28 | 7 | 12 | 21 | 19 |
| Dunnock | 5 | 7 | 5 | 5 | 12 | 3 | 6 | 5 | 4 |
| Robin | 8 | 8 | 7 | 4 | 15 | 5 | 11 | 10 | 7 |
| Wheatear | 0 | 0 | 0 | 0 | 1 | 1 | 0 | 0 | 0 |
| Song Thrush | 3 | 3 | 6 | 5 | 4 | 5 | 5 | 3 | 6 |
| Mistle Thrush | 0 | 4 | 4 | 0 | 0 | 1 | 2 | 5 | 0 |
| Blackbird | 8 | 11 | 11 | 11 | 10 | 7 | 9 | 10 | 8 |
| Whitethroat | 0 | 0 | 0 | 1 | 2 | 0 | 1 | 0 | 0 |
| Blackcap | 1 | 0 | 2 | 0 | 3 | 2 | 0 | 0 | 0 |
| Willow Warbler | 0 | 0 | 0 | 0 | 5 | 0 | 0 | 0 | 1 |
| Chiffchaff | 3 | 0 | 3 | 0 | 4 | 1 | 0 | 0 | 1 |
| Goldcrest | 3 | 5 | 4 | 2 | 3 | 2 | 2 | 5 | 1 |
| Spotted Flycatcher | 0 | 0 | 0 | 0 | 0 | 1 | 0 | 0 | 0 |
| Blue Tit | 6 | 11 | 7 | 9 | 9 | 4 | 4 | 5 | 7 |
| Coal Tit | 7 | 4 | 4 | 1 | 4 | 2 | 2 | 6 | 3 |
| Great Tit | 3 | 6 | 5 | 2 | 4 | 0 | 3 | 4 | 2 |
| Long-tailed Tit | 2 | 12 | 0 | 0 | 2 | 0 | 0 | 12 | 0 |
| Magpie | 2 | 3 | 5 | 2 | 2 | 4 | 3 | 5 | 2 |
| Jackdaw | 20 | 10 | 32 | 17 | 55 | 6 | 22 | 20 | 7 |
| Rook | 152 | 54 | 104 | 60 | 55 | 68 | 32 | 65 | 25 |
| Hooded Crow | 3 | 2 | 3 | 0 | 3 | 2 | 1 | 2 | 2 |
| Raven | 1 | 0 | 0 | 0 | 1 | 0 | 0 | 0 | 0 |
| Starling | 15 | 15 | 16 | 30 | 0 | 20 | 10 | 44 | 36 |
| House Sparrow | 10 | 2 | 1 | 3 | 10 | 0 | 0 | 1 | 3 |
| Tree Sparrow | 0 | 2 | 0 | 0 | 3 | 2 | 2 | 0 | 0 |
| Chaffinch | 10 | 14 | 7 | 5 | 10 | 4 | 12 | 7 | 10 |
| Greenfinch | 3 | 8 | 4 | 2 | 4 | 2 | 7 | 3 | 4 |
| Common Redpoll | 0 | 2 | 0 | 0 | 0 | 0 | 0 | 0 | 0 |
| Goldfinch | 2 | 6 | 5 | 3 | 2 | 0 | 3 | 1 | 3 |
| Linnet | 3 | 2 | 2 | 3 | 1 | 0 | 6 | 1 | 1 |
| Bullfinch | 2 | 2 | 0 | 0 | 1 | 0 | 0 | 4 | 2 |
| Reed Bunting | 0 | 1 | 0 | 1 | 2 | 0 | 0 | 0 | 0 |
| Yellowhammer | 3 | 4 | 0 | 1 | 12 | 4 | 10 | 0 | 0 |
| Grey Heron | 0 | 1 | 0 | 0 | 0 | 0 | 1 | 0 | 0 |
| Buzzard | 1 | 0 | 0 | 0 | 0 | 0 | 0 | 0 | 0 |
| Kestrel | 0 | 0 | 0 | 0 | 0 | 1 | 0 | 0 | 0 |
| Golden Plover | 0 | 0 | 0 | 0 | 0 | 0 | 1 | 0 | 0 |
| Swallow | 0 | 0 | 0 | 0 | 0 | 0 | 0 | 0 | 1 |
| Grey Wagtail | 0 | 3 | 0 | 0 | 0 | 0 | 0 | 0 | 1 |
| Grasshopper Warbler | 0 | 0 | 1 | 0 | 0 | 0 | 0 | 0 | 0 |
| Sedge Warbler | 0 | 0 | 0 | 0 | 0 | 0 | 0 | 1 | 2 |

Table S5: East Anglian farmland count data

|  | SiteA | SiteB | SiteC | SiteD | SiteE | SiteF | SiteG | SiteH | SiteI | SiteJ | SiteK | SiteL | SiteM | SiteN | SiteO | SiteP | SiteQ | SiteR | SiteS | SiteT |
| --- | --- | --- | --- | --- | --- | --- | --- | --- | --- | --- | --- | --- | --- | --- | --- | --- | --- | --- | --- | --- |
| Chaffinch | 27 | 41 | 67 | 47 | 74 | 63 | 53 | 45 | 56 | 71 | 42 | 38 | 60 | 60 | 44 | 66 | 72 | 70 | 75 | 44 |
| Robin | 8 | 10 | 24 | 11 | 25 | 24 | 5 | 15 | 15 | 35 | 9 | 16 | 26 | 19 | 10 | 11 | 37 | 34 | 36 | 21 |
| Wood Pigeon | 78 | 239 | 416 | 52 | 521 | 342 | 657 | 76 | 239 | 315 | 71 | 381 | 177 | 463 | 90 | 151 | 496 | 317 | 103 | 145 |
| Wren | 15 | 18 | 15 | 22 | 25 | 30 | 24 | 23 | 20 | 45 | 12 | 21 | 38 | 13 | 21 | 19 | 44 | 40 | 47 | 11 |
| Great Tit | 6 | 3 | 7 | 9 | 15 | 18 | 9 | 5 | 16 | 21 | 9 | 25 | 12 | 12 | 9 | 9 | 13 | 9 | 31 | 15 |
| Coal Tit | 1 | 1 | 1 | 1 | 2 | 0 | 0 | 0 | 0 | 0 | 0 | 1 | 0 | 0 | 0 | 0 | 1 | 0 | 0 | 2 |
| Greenfinch | 10 | 14 | 35 | 14 | 26 | 13 | 23 | 18 | 35 | 48 | 7 | 37 | 55 | 9 | 12 | 20 | 46 | 61 | 42 | 17 |
| Song Thrush | 4 | 3 | 5 | 4 | 9 | 3 | 3 | 7 | 6 | 9 | 3 | 4 | 5 | 4 | 7 | 6 | 9 | 5 | 6 | 6 |
| Blackbird | 16 | 13 | 47 | 25 | 50 | 19 | 18 | 23 | 44 | 70 | 14 | 34 | 39 | 27 | 32 | 31 | 64 | 60 | 63 | 18 |
| Dunnock | 12 | 7 | 20 | 13 | 16 | 14 | 14 | 11 | 21 | 24 | 6 | 25 | 17 | 22 | 12 | 19 | 14 | 16 | 14 | 12 |
| Blue Tit | 5 | 9 | 15 | 9 | 24 | 39 | 30 | 8 | 13 | 27 | 11 | 35 | 19 | 20 | 10 | 9 | 12 | 29 | 24 | 27 |
| Jackdaw | 13 | 7 | 15 | 22 | 2 | 30 | 6 | 11 | 25 | 30 | 62 | 32 | 7 | 29 | 3 | 11 | 11 | 15 | 74 | 34 |
| Carrion Crow | 3 | 18 | 9 | 4 | 10 | 46 | 57 | 3 | 11 | 9 | 2 | 17 | 26 | 15 | 10 | 4 | 15 | 14 | 13 | 17 |
| Chiffchaff | 1 | 5 | 1 | 4 | 4 | 11 | 2 | 4 | 3 | 6 | 3 | 6 | 5 | 1 | 3 | 2 | 5 | 4 | 7 | 1 |
| Bullfinch | 2 | 2 | 5 | 2 | 7 | 3 | 0 | 4 | 5 | 6 | 3 | 4 | 4 | 3 | 4 | 1 | 2 | 2 | 4 | 3 |
| Green Woodpecker | 2 | 2 | 2 | 1 | 2 | 2 | 1 | 2 | 0 | 1 | 3 | 5 | 2 | 2 | 1 | 3 | 2 | 3 | 4 | 4 |
| Goldcrest | 1 | 1 | 2 | 1 | 1 | 3 | 3 | 1 | 1 | 3 | 1 | 4 | 2 | 0 | 1 | 1 | 2 | 1 | 0 | 1 |
| Jay | 2 | 0 | 0 | 2 | 1 | 4 | 1 | 2 | 0 | 0 | 0 | 2 | 0 | 1 | 1 | 1 | 1 | 2 | 1 | 5 |
| Long-tailed Tit | 5 | 2 | 8 | 5 | 6 | 8 | 2 | 3 | 2 | 8 | 3 | 8 | 6 | 5 | 4 | 2 | 4 | 5 | 5 | 4 |
| Linnet | 10 | 6 | 38 | 8 | 29 | 29 | 29 | 6 | 32 | 25 | 4 | 46 | 18 | 35 | 15 | 10 | 10 | 26 | 29 | 22 |
| Rook | 26 | 118 | 153 | 440 | 70 | 84 | 11 | 21 | 11 | 69 | 112 | 45 | 4 | 120 | 29 | 30 | 50 | 107 | 224 | 88 |
| Skylark | 25 | 33 | 94 | 14 | 90 | 78 | 85 | 9 | 135 | 41 | 8 | 41 | 56 | 116 | 25 | 40 | 42 | 53 | 35 | 75 |
| Grey Partridge | 2 | 2 | 6 | 0 | 2 | 6 | 6 | 2 | 0 | 2 | 0 | 0 | 2 | 4 | 2 | 2 | 2 | 4 | 4 | 9 |
| Yellowhammer | 3 | 22 | 27 | 14 | 44 | 19 | 21 | 19 | 50 | 16 | 14 | 9 | 28 | 32 | 25 | 33 | 28 | 28 | 41 | 28 |
| Stock Dove | 8 | 1 | 4 | 6 | 5 | 7 | 7 | 8 | 3 | 3 | 6 | 6 | 10 | 14 | 5 | 6 | 12 | 5 | 7 | 7 |
| Goldfinch | 7 | 6 | 10 | 6 | 14 | 8 | 9 | 8 | 10 | 14 | 6 | 4 | 10 | 21 | 8 | 12 | 13 | 11 | 12 | 13 |
| Marsh Tit | 2 | 0 | 1 | 0 | 1 | 0 | 0 | 3 | 0 | 1 | 0 | 0 | 0 | 0 | 0 | 0 | 0 | 2 | 0 | 0 |
| Blackcap | 4 | 3 | 7 | 6 | 11 | 8 | 2 | 7 | 6 | 12 | 3 | 9 | 7 | 7 | 6 | 3 | 4 | 10 | 11 | 7 |
| Willow Warbler | 0 | 0 | 5 | 4 | 2 | 2 | 0 | 4 | 1 | 6 | 0 | 0 | 1 | 2 | 3 | 1 | 2 | 3 | 3 | 2 |
| Great Spotted Woodpecker | 1 | 1 | 1 | 2 | 6 | 3 | 1 | 1 | 2 | 4 | 1 | 4 | 3 | 2 | 0 | 1 | 2 | 1 | 4 | 0 |
| Mistle Thrush | 2 | 1 | 3 | 4 | 1 | 1 | 2 | 2 | 1 | 6 | 3 | 11 | 0 | 1 | 3 | 4 | 5 | 3 | 3 | 6 |
| Sparrowhawk | 1 | 0 | 0 | 1 | 1 | 0 | 0 | 1 | 0 | 1 | 2 | 0 | 0 | 0 | 1 | 0 | 0 | 1 | 1 | 1 |
| Moorhen | 0 | 2 | 1 | 1 | 2 | 8 | 8 | 2 | 2 | 8 | 0 | 11 | 6 | 1 | 2 | 1 | 6 | 10 | 2 | 5 |
| Starling | 8 | 2 | 13 | 15 | 4 | 23 | 23 | 3 | 20 | 107 | 6 | 18 | 12 | 9 | 9 | 14 | 9 | 17 | 12 | 9 |
| Collared Dove | 5 | 4 | 6 | 2 | 13 | 3 | 6 | 2 | 22 | 18 | 1 | 26 | 14 | 5 | 7 | 7 | 24 | 16 | 11 | 5 |
| Whitethroat | 6 | 19 | 28 | 13 | 25 | 18 | 4 | 25 | 48 | 20 | 18 | 17 | 49 | 14 | 17 | 11 | 25 | 31 | 44 | 23 |
| Kestrel | 2 | 1 | 1 | 3 | 1 | 2 | 2 | 2 | 1 | 2 | 2 | 3 | 1 | 2 | 2 | 1 | 1 | 4 | 1 | 2 |
| Meadow Pipit | 0 | 5 | 0 | 0 | 0 | 1 | 9 | 0 | 0 | 0 | 0 | 2 | 0 | 6 | 0 | 1 | 0 | 0 | 0 | 3 |
| Pied Wagtail | 6 | 6 | 6 | 1 | 1 | 8 | 13 | 4 | 3 | 3 | 4 | 2 | 3 | 3 | 5 | 4 | 10 | 4 | 3 | 2 |
| House Sparrow | 20 | 7 | 19 | 5 | 46 | 4 | 6 | 4 | 33 | 45 | 2 | 1 | 28 | 2 | 7 | 21 | 39 | 40 | 14 | 0 |
| Lapwing | 5 | 37 | 15 | 0 | 0 | 36 | 36 | 12 | 10 | 12 | 0 | 7 | 0 | 1 | 6 | 6 | 23 | 2 | 3 | 6 |
| Magpie | 3 | 2 | 1 | 7 | 4 | 12 | 4 | 5 | 8 | 4 | 3 | 8 | 13 | 4 | 5 | 2 | 1 | 4 | 4 | 0 |
| Treecreeper | 1 | 0 | 0 | 0 | 0 | 1 | 0 | 0 | 0 | 0 | 0 | 1 | 0 | 0 | 0 | 0 | 0 | 0 | 0 | 0 |
| Little Owl | 0 | 2 | 0 | 1 | 1 | 2 | 2 | 2 | 1 | 0 | 1 | 1 | 1 | 0 | 2 | 1 | 1 | 1 | 1 | 0 |
| Swallow | 8 | 12 | 10 | 7 | 26 | 15 | 15 | 9 | 6 | 16 | 8 | 13 | 20 | 19 | 6 | 8 | 12 | 17 | 19 | 14 |
| Sand Martin | 0 | 0 | 0 | 0 | 0 | 0 | 0 | 0 | 0 | 0 | 0 | 3 | 0 | 0 | 0 | 6 | 0 | 0 | 0 | 0 |
| Wheatear | 0 | 0 | 2 | 0 | 0 | 0 | 3 | 0 | 0 | 0 | 0 | 0 | 0 | 1 | 1 | 1 | 1 | 0 | 2 | 2 |
| Snipe | 0 | 0 | 0 | 0 | 0 | 0 | 1 | 0 | 0 | 0 | 0 | 0 | 0 | 0 | 0 | 0 | 0 | 0 | 0 | 0 |
| Golden Plover | 0 | 0 | 1 | 0 | 0 | 0 | 140 | 0 | 0 | 0 | 0 | 0 | 0 | 0 | 0 | 0 | 0 | 0 | 0 | 0 |
| Lesser Whitethroat | 4 | 5 | 5 | 4 | 10 | 10 | 1 | 8 | 13 | 8 | 5 | 4 | 9 | 4 | 4 | 7 | 9 | 6 | 6 | 3 |
| Feral Pigeon | 0 | 0 | 3 | 60 | 0 | 0 | 0 | 0 | 0 | 0 | 0 | 0 | 0 | 0 | 0 | 2 | 4 | 2 | 1 | 0 |
| Corn Bunting | 0 | 0 | 0 | 0 | 0 | 0 | 0 | 0 | 0 | 0 | 0 | 0 | 0 | 19 | 0 | 0 | 0 | 0 | 0 | 0 |
| Nuthatch | 0 | 0 | 0 | 1 | 0 | 0 | 0 | 0 | 0 | 0 | 0 | 0 | 0 | 0 | 0 | 0 | 0 | 0 | 0 | 0 |
| Reed Bunting | 0 | 0 | 3 | 0 | 1 | 1 | 1 | 0 | 2 | 1 | 0 | 0 | 3 | 4 | 0 | 0 | 1 | 0 | 1 | 1 |
| Grey Heron | 3 | 1 | 0 | 3 | 0 | 3 | 0 | 1 | 1 | 1 | 1 | 2 | 2 | 2 | 1 | 0 | 0 | 1 | 0 | 0 |
| Garden Warbler | 0 | 1 | 1 | 1 | 1 | 0 | 0 | 1 | 1 | 1 | 1 | 0 | 1 | 1 | 0 | 1 | 2 | 0 | 2 | 2 |
| Turtle Dove | 0 | 6 | 4 | 2 | 2 | 1 | 0 | 16 | 1 | 7 | 1 | 1 | 8 | 2 | 1 | 8 | 5 | 4 | 10 | 1 |
| House Martin | 15 | 10 | 28 | 13 | 36 | 2 | 2 | 4 | 52 | 14 | 10 | 13 | 3 | 20 | 5 | 15 | 2 | 2 | 16 | 4 |
| Swift | 3 | 12 | 6 | 20 | 11 | 7 | 7 | 6 | 2 | 20 | 14 | 4 | 6 | 24 | 5 | 14 | 44 | 42 | 7 | 6 |
| Cuckoo | 0 | 0 | 0 | 0 | 1 | 4 | 4 | 2 | 2 | 2 | 0 | 0 | 2 | 0 | 2 | 1 | 2 | 2 | 1 | 0 |
| Grasshopper Warbler | 0 | 0 | 0 | 0 | 1 | 0 | 0 | 0 | 0 | 0 | 0 | 0 | 0 | 0 | 0 | 0 | 0 | 0 | 0 | 0 |
| Yellow Wagtail | 1 | 2 | 0 | 0 | 1 | 1 | 1 | 0 | 2 | 0 | 1 | 0 | 1 | 2 | 1 | 1 | 0 | 1 | 0 | 0 |
| Buzzard | 0 | 0 | 1 | 1 | 1 | 0 | 0 | 1 | 1 | 0 | 0 | 0 | 0 | 1 | 0 | 1 | 2 | 1 | 0 | 0 |
| Oystercatcher | 1 | 0 | 0 | 0 | 0 | 2 | 2 | 0 | 0 | 1 | 4 | 1 | 0 | 0 | 2 | 4 | 3 | 0 | 0 | 0 |
| Spotted Flycatcher | 2 | 1 | 0 | 0 | 0 | 2 | 2 | 2 | 0 | 0 | 1 | 0 | 0 | 0 | 0 | 1 | 1 | 1 | 0 | 0 |
| Tawny Owl | 0 | 0 | 0 | 0 | 0 | 1 | 1 | 2 | 0 | 0 | 0 | 0 | 0 | 0 | 0 | 0 | 0 | 0 | 0 | 0 |
| Sedge Warbler | 0 | 0 | 0 | 0 | 0 | 0 | 0 | 0 | 1 | 0 | 0 | 0 | 0 | 1 | 0 | 0 | 1 | 0 | 1 | 1 |
| Tree Sparrow | 0 | 0 | 0 | 0 | 0 | 0 | 0 | 0 | 0 | 0 | 0 | 0 | 0 | 1 | 4 | 11 | 0 | 0 | 0 | 0 |
| Nightingale | 0 | 0 | 0 | 0 | 0 | 0 | 0 | 0 | 0 | 0 | 1 | 0 | 0 | 0 | 0 | 0 | 0 | 0 | 0 | 0 |
| Marsh Harrier | 0 | 0 | 0 | 0 | 0 | 0 | 0 | 1 | 0 | 0 | 0 | 0 | 0 | 0 | 0 | 1 | 3 | 0 | 0 | 0 |
| Barn Owl | 0 | 1 | 0 | 0 | 0 | 0 | 0 | 1 | 0 | 1 | 0 | 0 | 0 | 0 | 0 | 0 | 2 | 1 | 0 | 0 |
| Grey Wagtail | 1 | 0 | 0 | 0 | 0 | 7 | 0 | 0 | 0 | 0 | 0 | 5 | 0 | 0 | 0 | 0 | 0 | 0 | 0 | 0 |
| Marsh Sandpiper | 0 | 0 | 0 | 0 | 0 | 0 | 0 | 0 | 0 | 0 | 0 | 0 | 0 | 1 | 0 | 0 | 0 | 0 | 0 | 0 |
| Hobby | 0 | 0 | 0 | 0 | 0 | 0 | 0 | 0 | 0 | 1 | 0 | 0 | 1 | 0 | 1 | 0 | 0 | 0 | 0 | 0 |
| Curlew | 0 | 0 | 0 | 0 | 0 | 0 | 0 | 0 | 0 | 0 | 0 | 0 | 0 | 0 | 0 | 0 | 2 | 0 | 0 | 0 |
| Firecrest | 0 | 1 | 0 | 0 | 0 | 0 | 0 | 0 | 0 | 0 | 0 | 0 | 0 | 0 | 0 | 0 | 0 | 0 | 0 | 0 |
| Fieldfare | 0 | 0 | 0 | 0 | 0 | 0 | 5 | 0 | 0 | 0 | 0 | 0 | 0 | 0 | 0 | 0 | 0 | 0 | 0 | 0 |
| Reed Warbler | 0 | 0 | 0 | 1 | 0 | 0 | 0 | 0 | 0 | 0 | 0 | 0 | 0 | 0 | 0 | 0 | 0 | 0 | 0 | 0 |

Code S6: Diversity profiles considering breeding bird assemblages in Ireland and Britain

---

title: "Diversity Profiles- Island Scale"

output: html_document

---

##setup

```{r}

library(ape)

```

##load data

```{r}

trees <- read.nexus("allspp10000trees2.nex")

species <- length(trees[[1]]$tip.label)

totaltrees <- length(trees)

roi <- read.csv("ROIwithC.csv", header = TRUE, sep = ",")

gb <- read.csv("GBwithC.csv", header = TRUE, sep = ",")

ni <- read.csv("NIwithC.csv", header = TRUE, sep = ",")

regions <- 2

remove <- read.csv("Remove_Island_Scale.csv", header = TRUE, sep = ",")

namesorder <- read.csv("Names_Order_Island_Scale.csv", header = FALSE, sep = ",")

col.order <- namesorder[,1]

row.order <- col.order

btocodes <- namesorder[,2]

```

##calculate mean distances between nodes

```{r}

##generate a list of distance matrices for all trees

distlist <- list()

for (i in 1:totaltrees) {

distlist[[i]] <- cophenetic.phylo(trees[[i]])

}

##reorder matrices so data matches

for (i in 1:totaltrees) {

temp <- distlist[[i]]

temp <- temp[,col.order]

distlist[[i]] <- temp

}

for (i in 1:totaltrees) {

temp <- distlist[[i]]

temp <- temp[row.order,]

distlist[[i]] <- temp

}

##calculate mean distances across all 10,000 trees and standardize from 0-1

meandists <- Reduce("+", distlist)

meandists <- meandists/totaltrees

stand <- 1 - meandists/max(meandists)

##populate similarity matrix Z

Z <- stand

rownames(Z) <- btocodes

colnames(Z) <- btocodes

```

##remove species not included in the assemblage from Z

```{r}

removebto <- sort(remove$BTO)

temp <- c()

for (i in 1:length(removebto)) {

for(k in 1:species){

if(all.equal(colnames(Z)[k], removebto[i]) == T){

temp <- c(temp, k)

}

}

}

Z <- Z[-c(temp), -c(temp)]

species <- length(Z[1,])

```

##combine RoI and NI abundance data

```{r}

ireland <- roi[,2]+ni[,2]

names(ireland) <- roi[,1]

##change LR to FR for similarity matrix

names(ireland)[which(names(ireland) == "LR")] <- "FR"

temp <- c()

for(i in 1:species){

for(j in 1:species){

if(all.equal(names(ireland)[j], rownames(Z)[i]) == T){

temp[i] <- ireland[j]

}

}

}

ireland <- temp

names(ireland) <- colnames(Z)

```

##transform abundance estimates into relative abundances

```{r}

##initialize matrix

p <- matrix(data = 0, nrow = species, ncol = regions)

colnames(p) <- c("Ireland", "GB")

rownames(p) <- rownames(Z)

##ireland

pie <- ireland

for (i in 1:species) {

p[i,1] <- (ireland[i])/(sum(ireland))

}

##Britain

pgb <- gb[,2]

names(pgb) <- gb[,1]

names(pgb)[which(names(pgb) == "LR")] <- "FR"

temp<- c()

for(i in 1:species){

for(j in 1:species){

if(all.equal(names(pgb)[j], rownames(Z)[i]) == T){

temp[i] <- pgb[j]

}

}

}

pgb <- temp

names(pgb) <- colnames(Z)

for (i in 1:species) {

p[i,2] <- (pgb[i]/sum(pgb))

}

```

##Zp matrix (see Leinster & Cobbold eq. 1.2)

```{r}

##initialize matrix to 0

Zp <- matrix(0, species, regions)

colnames(Zp) <- colnames(p)

rownames(Zp) <- rownames(Z)

##populate Zp matrix

for (k in 1:regions) {

for (i in 1:species) {

for(j in 1:species){

Zp[i,k] <- Zp[i,k] + Z[i,j]*p[j,k]

}

}

}

```

##diversity matrix (see Leinster & Cobbold eq. 1.1)

```{r}

##set q values to 0-10.1 by 0.101

qvals <- seq(length = 101, from = 0, by = .101)

##initialize matrix to 0

Dqz <- matrix(0, length(qvals), regions)

colnames(Dqz) <- colnames(Zp)

##populate diversity matrix with diversity of order q for each q value (i) in

##each region (k)

for (k in 1:regions) {

for(iq in 1:length(qvals)) {

q <- qvals[iq]

for(zpi in 1:species){

if(Zp[zpi,k]>0){

Dqz[iq,k]<-Dqz[iq,k]+ p[zpi,k]*(Zp[zpi,k])^(q-1)

}

}

Dqz[iq,k] <- Dqz[iq,k]^(1/(1-q))

}

}

```

##plots

```{r}

##diversity profiles

plot(qvals, Dqz[,1], ylim = c(1.7,2.1), col = "orange", type = "l", lwd = 3,

xlab = "q", ylab = "Diversity of Order q")

points(qvals, Dqz[,2], col = "blue", type = "l", lwd =3, lty = 2)

legend("topright", c("Irish Assemblage Diversity", "British Assemblage Diversity"),

col = c("orange", "blue"), lty = c(1, 2), lwd = 3, bty = "n")

maintitle <- "Diversity Profiles"

subtitle <- "Irish and British Assemblages of 49 Common Breeding Species"

mtext(line = 1, maintitle)

mtext(line = 0, subtitle)

##vertical distance between profiles for each value q

distdqz <- Dqz[,2] - Dqz[,1]

plot(qvals,distdqz,xlab = "q", ylab = "Distance Between Profiles", pch = 20)

maintitle <- "Diversity Profile Similarity"

subtitle <- "Irish and British Assemblages of 49 Common Breeding Species"

mtext(line = 1, maintitle)

mtext(line = 0, subtitle)

##slopes

slopes <- Dqz

for (k in 1:regions) {

for (i in 2:length(qvals)) {

slopes[i,k] <-Dqz[i,k] - Dqz[i-1,k]

}

}

```

Code S7: Diversity profiles considering breeding bird assemblages on Irish and East Anglian farmland

---

title: "Diversity Profiles- Farmland Scale"

author: "Camille Groh"

output: html_document

---

##setup

```{r}

library(ape)

```

##load data

```{r}

trees <- read.nexus("allspp10000trees2.nex")

totaltrees <- length(trees)

species <- length(trees[[1]]$tip.label)

iecounts <- read.csv("IrelandMaxCountsFixed.csv", header = T, row.names = 1)

eacounts <- read.csv("EAMaxCountsFixed.csv", header = T, row.names = 1)

regions <- 2

namesorder <- read.csv("namesorder2.csv", header = F)

col.order <- namesorder[,1]

row.order <- col.order

btocodes <- namesorder[,2]

remove <- read.csv("removegame.csv", header = T, sep = ",")

```

##calculate mean distances between nodes, standardize, and populate similarity

##matrix

```{r}

##generate a list of distance matrices for all trees

distlist <- list()

for (i in 1:totaltrees) {

distlist[[i]] <- cophenetic.phylo(trees[[i]])

}

##reorder matrices so species positioning matches

for (i in 1:totaltrees) {

temp <- distlist[[i]]

temp <- temp[,col.order]

distlist[[i]] <- temp

}

for (i in 1:totaltrees) {

temp <- distlist[[i]]

temp <- temp[row.order,]

distlist[[i]] <- temp

}

##calculate mean distances across all 10,000 trees and standardize from 0-1

sumdists <- Reduce("+", distlist)

meandists <- sumdists/totaltrees

stand <- 1 - meandists/max(meandists)

##populate similarity matrix Z

Z <- stand

rownames(Z) <- btocodes

colnames(Z) <- btocodes

```

##remove extraneous species

```{r}

removebto <- remove$BTO

removebto <- sort(removebto)

##remove species from Z

temp <- c()

for (i in 1:length(removebto)) {

for(k in 1:species){

if(all.equal(colnames(Z)[k], removebto[i]) == T){

temp <- c(temp, k)

}

}

}

Z <- Z[-c(temp), -c(temp)]

species <- length(Z[1,])

```

##abundance matrix p

```{r}

##Irish count data to relative abundances

totals <- rowSums(iecounts)

pie <- totals/sum(totals)

##East Anglian count data to relative abundances

totals <- rowSums(eacounts)

pea <- totals/sum(totals)

##initialize abundance matrix p

allspp <- sort(unique(c(names(pie), names(pea))))

p <- matrix(0, length(allspp), regions)

rownames(p) <- allspp

colnames(p) <- c("Ireland", "East Anglia")

##populate p with Irish data

for(i in 1:length(allspp)){

for(j in 1:length(allspp)){

if(all.equal(rownames(p)[i], names(pie)[j]) == T){

p[i,1] <- pie[j]

}

}

}

##populate p with East Anglian data

for(i in 1:length(allspp)){

for(j in 1:length(allspp)){

if(all.equal(rownames(p)[i], names(pea)[j]) == T){

p[i,2] <- pea[j]

}

}

}

##combine HC and C

p[which(rownames(p) == "C"), 1] <- p[which(rownames(p) == "HC"), 1]

p <- p[-c(which(rownames(p) == "HC")),]

```

##Zp matrix

```{r}

##initialize matrix

Zp <- matrix(0, species, regions)

rownames(Zp) <- rownames(p)

colnames(Zp) <- colnames(p)

##populate matrix

for (k in 1:regions) {

for (i in 1:species) {

for(j in 1:species){

Zp[i,k] <- Zp[i,k] + Z[i,j]*p[j,k]

}

}

}

```

##diversity matrix (Dqz)

```{r}

##set q values to 0-10.1 by 0.101

qvals <- seq(length = 101, from = 0, by = .101)

##initialize matrix

Dqz <- matrix(0, length(qvals), regions)

colnames(Dqz) <- colnames(p)

rownames(Dqz) <- qvals

##populate diversity matrix with diversity of order q for each q value in each

##each region/year combination

for (k in 1:regions) {

for(iq in 1:length(qvals)) {

q <- qvals[iq]

for(zpi in 1:length(Zp[,k])){

if(Zp[zpi,k]>0){

Dqz[iq,k]<-Dqz[iq,k]+ p[zpi,k]*(Zp[zpi,k])^(q-1)

}

}

Dqz[iq,k] <- Dqz[iq,k]^(1/(1-q))

}

}

```

##plots

```{r}

##profiles

plot(qvals, Dqz[,1], type = "l", lwd = 3, lty = 1, col = "orange",

xlab = "q", ylab = "Diversity of Order q", ylim = c(1.75, 3.75))

points(qvals, Dqz[,2], type = "l", lty = 2, lwd = 3, col = "blue")

legend("topright", c("Irish Assemblage Diversity", "East Anglian Assemblage Diversity"),

lty = c(1,2), col = c("orange", "blue"),lwd = 3, bty = "n")

maintitle <- "Diversity Profiles"

subtitle <- "Irish and East Anglian Farmland Assemblages"

mtext(line = 1, maintitle)

mtext(line = 0, subtitle)

##distance

vertdist = Dqz[,2] - Dqz[,1]

plot(qvals, vertdist, pch = 20, xlab = "q",

ylab = "Vertical Distance Between Diversity Profiles", ylim = c(0,1.5))

maintitle <- "Diversity Profile Similarity"

subtitle <- "Irish and East Anglian Farmland Assemblages"

mtext(line = 1, maintitle)

mtext(line = 0, subtitle)

```

Code S8: Diversity profiles considering breeding bird assemblages of overlapping species on Irish and East Anglian farmland

---

title: "Diversity Profiles- Farmland Overlapping Species"

author: "Camille Groh"

output: html_document

---

##setup

```{r}

library(ape)

```

##load data

```{r}

trees <- read.nexus("allspp10000trees2.nex")

totaltrees <- length(trees)

species <- length(trees[[1]]$tip.label)

iecounts <- read.csv("IrelandMaxCountsFixed.csv", header = T, row.names = 1)

eacounts <- read.csv("EAMaxCountsFixed.csv", header = T, row.names = 1)

regions <- 2

namesorder <- read.csv("namesorder2.csv", header = F)

col.order <- namesorder[,1]

row.order <- col.order

btocodes <- namesorder[,2]

remove <- read.csv("removeoverlap.csv", header = T, sep = ",")

```

##calculate mean distances between nodes, standardize, and populate similarity

##matrix

```{r}

##generate a list of distance matrices for all trees

distlist <- list()

for (i in 1:totaltrees) {

distlist[[i]] <- cophenetic.phylo(trees[[i]])

}

##reorder matrices so species positioning matches

for (i in 1:totaltrees) {

temp <- distlist[[i]]

temp <- temp[,col.order]

distlist[[i]] <- temp

}

for (i in 1:totaltrees) {

temp <- distlist[[i]]

temp <- temp[row.order,]

distlist[[i]] <- temp

}

##calculate mean distances across all 10,000 trees and standardize from 0-1

sumdists <- Reduce("+", distlist)

meandists <- sumdists/totaltrees

stand <- 1 - (meandists/(max(meandists)))

##populate similarity matrix Z

Z <- stand

rownames(Z) <- btocodes

colnames(Z) <- btocodes

```

##remove extraneous species

```{r}

removebto <- remove$BTO

removebto <- sort(removebto)

##remove species from Irish data

temp <- c()

for (i in 1:length(removebto)) {

for(k in 1:length(iecounts[ ,1])){

if(all.equal(rownames(iecounts)[k], removebto[i]) == T){

temp <- c(temp, k)

}

}

}

iecounts <- iecounts[-c(temp),]

##remove species from East Anglia data

temp <- c()

for (i in 1:length(removebto)) {

for(k in 1:length(eacounts[ ,1])){

if(all.equal(rownames(eacounts)[k], removebto[i]) == T){

temp <- c(temp, k)

}

}

}

eacounts <- eacounts[-c(temp),]

##remove species from Z

temp <- c()

for (i in 1:length(removebto)) {

for(k in 1:species){

if(all.equal(colnames(Z)[k], removebto[i]) == T){

temp <- c(temp, k)

}

}

}

Z <- Z[-c(temp), -c(temp)]

species <- length(Z[1,])

```

##abundance matrix p

```{r}

##Irish count data to relative abundances

totals <- rowSums(iecounts)

pie <- totals/sum(totals)

##East Anglian count data to relative abundances

totals <- rowSums(eacounts)

pea <- totals/sum(totals)

##initialize abundance matrix p

allspp <- sort(unique(c(names(pie), names(pea))))

p <- matrix(0, length(allspp), regions)

rownames(p) <- allspp

colnames(p) <- c("Ireland", "East Anglia")

##populate p with Irish data

for(i in 1:length(allspp)){

for(j in 1:length(allspp)){

if(all.equal(rownames(p)[i], names(pie)[j]) == T){

p[i,1] <- pie[j]

}

}

}

##populate p with East Anglia data

for(i in 1:length(allspp)){

for(j in 1:length(allspp)){

if(all.equal(rownames(p)[i], names(pea)[j]) == T){

p[i,2] <- pea[j]

}

}

}

##combine HC and C

p[which(rownames(p) == "C"), 1] <- p[which(rownames(p) == "HC"), 1]

p <- p[-c(which(rownames(p) == "HC")),]

```

##Zp matrix

```{r}

##initialize matrix

Zp <- matrix(0, species, regions)

rownames(Zp) <- rownames(p)

colnames(Zp) <- colnames(p)

##populate matrix

for (k in 1:regions) {

for (i in 1:species) {

for(j in 1:species){

Zp[i,k] <- Zp[i,k] + Z[i,j]*p[j,k]

}

}

}

```

##diversity matrix (Dqz)

```{r}

##set q values to 0-10.1 by 0.101

qvals <- seq(length = 101, from = 0, by = .101)

##initialize matrix

Dqz <- matrix(0, length(qvals), regions)

colnames(Dqz) <- colnames(p)

rownames(Dqz) <- qvals

##populate diversity matrix with diversity of order q for each q value in each

##each region/year combination

for (k in 1:regions) {

for(iq in 1:length(qvals)) {

q <- qvals[iq]

for(zpi in 1:species){

if(Zp[zpi,k]>0){

Dqz[iq,k]<-Dqz[iq,k]+ p[zpi,k]*(Zp[zpi,k])^(q-1)

}

}

Dqz[iq,k] <- Dqz[iq,k]^(1/(1-q))

}

}

```

##plots

```{r}

##profiles

plot(qvals, Dqz[,1], type = "l", lwd = 3, lty = 1, col = "orange",

xlab = "q", ylab = "Diversity of Order q", ylim = c(1.75, 2.5))

points(qvals, Dqz[,2], type = "l", lty = 2, lwd = 3, col = "blue")

legend("topright", c("Irish Assemblage Diversity", "East Anglian Assemblage Diversity"),

lty = c(1,2), col = c("orange", "blue"),lwd = 3, bty = "n")

maintitle <- "Diversity Profiles"

subtitle <- "Irish and East Anglian Overlapping Farmland Assemblages"

mtext(line = 1, maintitle)

mtext(line = 0, subtitle)

##distance

vertdist = abs(Dqz[,2] - Dqz[,1])

plot(qvals, vertdist, pch = 20, xlab = "q",

ylab = "Vertical Distance Between Diversity Profiles")

maintitle <- "Diversity Profile Similarity"

subtitle <- "Irish and East Anglian Overlapping Farmland Assemblages"

mtext(line = 1, maintitle)

mtext(line = 0, subtitle)

```
